# A hybrid differential evolution based on gaining‑sharing knowledge algorithm and harris hawks optimization

**DOI:** 10.1371/journal.pone.0250951

**Published:** 2021-04-30

**Authors:** Xuxu Zhong, Meijun Duan, Xiao Zhang, Peng Cheng

**Affiliations:** 1 National Key Laboratory of Fundamental Science on Synthetic Vision, Sichuan University, Chengdu, China; 2 School of Computer and Software Engineering, Xihua University, Chengdu, China; 3 School of Aeronautics and Astronautics, Sichuan University, Chengdu, China; Torrens University Australia, AUSTRALIA

## Abstract

Differential evolution (DE) is favored by scholars for its simplicity and efficiency, but its ability to balance exploration and exploitation needs to be enhanced. In this paper, a hybrid differential evolution with gaining-sharing knowledge algorithm (GSK) and harris hawks optimization (HHO) is proposed, abbreviated as DEGH. Its main contribution lies are as follows. First, a hybrid mutation operator is constructed in DEGH, in which the two-phase strategy of GSK, the classical mutation operator “rand/1” of DE and the soft besiege rule of HHO are used and improved, forming a double-insurance mechanism for the balance between exploration and exploitation. Second, a novel crossover probability self-adaption strategy is proposed to strengthen the internal relation among mutation, crossover and selection of DE. On this basis, the crossover probability and scaling factor jointly affect the evolution of each individual, thus making the proposed algorithm can better adapt to various optimization problems. In addition, DEGH is compared with eight state-of-the-art DE algorithms on 32 benchmark functions. Experimental results show that the proposed DEGH algorithm is significantly superior to the compared algorithms.

## 1 Introduction

Whether in the field of science or engineering, problem optimization is a hot topic. Many researchers are keen to use meta-heuristic algorithms to solve optimization problems, leading to the emergence of various meta-heuristic algorithms, such as Evolution strategies (ES) [1], genetic algorithm (GA) [2], differential evolution [3], particle swarm optimization (PSO) [4], artificial bee colony (ABC) [5], gravitational search algorithm (GSA) [6], teaching–learning-based optimization (TLBO) [7], moth-flame optimization (MFO) [8], whale optimization algorithm (WOA) [9], harris hawks optimization (HHO) [10] and gaining-sharing knowledge algorithm (GSK) [11].

Since its inception, differential evolution (DE) has become one of the most commonly used meta-heuristic algorithms for solving optimization problems [12]. Many scholars have improved DE and applied it in diverse fields, such as clinical medicine [13], text classification [14], optics [15], energy [16] and neural network [17]. Improvements studies to DE can be divided into two broad categories: 1) Changes of DE compositions, which enhance the performance of the original DE by improving the mutation, crossover, selection operation and adjusting control parameters; 2) hybrid DE with other meta-heuristic algorithms to improve performance by combing their respective advantages.

At each generation, the evolution of individuals in differential evolution mainly goes through three stages: mutation, crossover and selection. These stages are the critical targets for the improvements of DE components, among which the mutation operation is the most important. Zhang and Sanderson [18] proposed the famous “*DE*/*current*−*to*−*pbest*/1” mutation operator in their proposed adaptive DE algorithm (JADE), which improved the mutation by using the first 100*p*% individuals and an external archive containing suboptimal individuals. Wang et al. [19] facilitate a self-adaptive differential evolution algorithm with improved mutation mode (IMMSADE), which ameliorate the classic mutation operator “*DE*/*rand*/1” by attaching a benchmark factor to the basis vector. Zheng et al. [20] proposed a collective information-powered DE (CIPDE), a collective individual contained in the mutation operator of which is a linear combination of *m* individuals with optimal fitness values. Mohamed et al. [21] proposed two enhanced DE variants (EBDE and EDE), in which three different individuals were ranked to participate in mutations, the difference being that the former’s individuals were randomly selected from the top *p* individuals and from the entire population, while the latter three individuals were all randomly chosen from the population. Li et al. [22] presented an improved differential evolution algorithm with dual mutation strategies collaboration (DMCDE), which applied the improved DE/*rand*/2 and DE/*best*/2 based on an elite guidance mechanism. Ghosh et al. [23] proposed a switched parameter DE, in which each individual randomly selected binary crossover operator or *BLX*−*α*−*β* crossover operator. Tian et al. [24] presented a DE with improved individual-based parameter setting and selection strategy (IDEI), which developed a diversity selection strategy based on the newly defined weighted fitness value. Cheng et al. [25] proposed an improved DE with fitness and diversity ranking-based mutation operator (FDDE), which judged the contribution of individuals participating in the "DE/rand/1" mutation strategy to population diversity according to their fitness values, and rearranged the positions of the three random individuals based on the ranking information of individual diversity and fitness values.

The control parameters of DE include population size *NP*, scaling factor *F* and crossover probability *CR*, which are the other direction of improvements of DE components. Tanabe and Fukunaga [26] proposed a success-history based parameter adaptation for DE (SHADE). By establishing new historical storages *M*_*cr*_ and *M*_*f*_, *CR* and *F* with good performance in the past were preserved, and new parameter pairs were sampled from them. Shortly after that, Tanabe and Fukunaga [27] raised an enhanced version that added a population size reduction rule to the SHADE (LSHADE). After the end of each evolutionary process, the population size of the next generation was reduced by a linear function. Poláková et al. [28] described a new mechanism of population size adaption to DE, which evaluated the current population diversity based on European distance and adjusted the *NP* size according to the evaluation results. Meng et al. [29] put forward a DE variant with novel control parameter adaptation (PaDE), which included a grouping strategy for adjusting *F* and *CR* and a parabolic reduction rule for changing *NP*. Li et al. [30] proposed an enhanced adaptive ED algorithm (EJADE), which introduced a crossover probability sorting mechanism and dynamic population reduction strategy based on JADE. Wang et al. [31] proposed a self-adaptive ensemble-based DE (SAEDE), which set the control parameters of each generation through self-adaptive and integration mechanisms, reducing the need for user setting. Xue and Chen introduced [32] an adaptive compact DE (ACDE), in which F and CR obeyed the Cauchy distribution and uniform distribution respectively and were adaptively adjusted according to their respective weighted Lehmer means.

Compared with component improvement, the study of mixing with other algorithms to improve the performance of DE is more novel, which often integrates the advantages of DE and different meta-heuristic algorithms. Guo et al. [33] presented an enhanced self-adaptive DE (ESADE) that combined simulated annealing in the selection stage. By comparing ESADE with the version without simulated annealing, the experimental results showed that ESADE with simulated annealing had better global search capability. Jadon et al. [34] proposed a hybrid artificial bee colony with DE (HABCDE), which applied DE to the onlooker bee stage of the ABC algorithm for faster convergence. Mohamed et al. [35] introduced a semi-parametric adaptation method in the LSHADE hybridized with covariance matrix adaptation evolution strategy (LSHADE-SPACMA), where the crossover operation of DE was applied to the covariance matrix adaptation evolution strategy improve the exploration capability. Zhao et al. [36] proposed a hybrid algorithm based on self-adaptive gravitational search algorithm and DE (SGSADE), which introduced the mutation and crossover of DE into the GSA, improved the local search ability and prevented the rapid loss of population diversity. A hybrid algorithm for DE and particle swarm optimization (DEPSO) was proposed by Wang et al. [37]. At each generation of DEPSO, each individual was determined by a selection factor whether to adopt the improved *rand*/1 mutation operator or the PSO mutation operator. Luo and Shi [38] mixed a modified DE with whale optimization algorithm (MDE-WOA), which took advantage of the modified DE strong searching ability to avoid WOA falling into local optimal and increased population diversity. Li et al. [39] proposed a hybrid adaptive teaching–learning-based optimization with DE (ATLDE), which embed DE into the learning stage of TLBO. The population of a hybrid symbiotic DE moth-flame optimization algorithm (HSDE-MFO) proposed by Wu et al. [40] was divided into two groups, which were used for exploration-oriented DE strategy and exploitation-oriented MFO strategy respectively. Taking advantage of the ease implementation of boltzmann annealing algorithm [41] and the good diversity of solutions and effective iteration process of DE, Li et al. [42] proposed a modified boltzmann annealing Differential Evolution algorithm (BADE). Ahmadianfar et al. [43] proposed an adaptive DE with PSO (A-DEPSO), which utilized PSO to improve the mutation operation of DE to promote the global search ability and accelerate convergence, and introduced a crossover probability adaptation rate in the crossover operation of DE to increase the local search capability.

Although the performance of DE has been enhanced by the methods mentioned above, some inherent problems are still worth pondering. First of all, whether it is the improvement of DE components or hybridization of DE with other meta-heuristic algorithms, the mutation, crossover, and selection steps of these methods are relatively independent, and the internal connection of DE framework is not high. Secondly, for the research of hybrid improvement, most of them are based on the combination of DE and a certain meta-heuristic algorithm. Not only the meta-heuristic algorithm used is not novel enough, but also the balance between exploration and exploitation is considered in a single way. Therefore, a hybrid differential evolution algorithm based on gaining-sharing knowledge algorithm and harris hawks optimization (DEGH) is proposed in this paper.

The rest of this paper is structured as follows. Section 2 covers the basics of differential evolution (DE), gaining-sharing knowledge algorithm (GSK), and harris hawks optimization (HHO). Section 3 introduces the proposed DEGH in detail. Section 4 presents a series of experimental results and analyses. Section 5 summaries the whole paper and puts forward the research direction in the future.

## 2 Preliminaries

This section describes the basic principles of differential evolution (DE), gaining-sharing knowledge algorithm (GSK) and harris hawks optimization (HHO).

### 2.1. Differential evolution

The framework structure of DE mainly includes four stages: initialization, mutation, crossover and selection, among which the last three stages are the cyclic evolution process based on the population.

#### 2.1.1 Initialization

For a minimization problem *minf*(*X*), the population *P*_g_ in DE can be defined as:

$$\left\{ \begin{array}{c} P_{g}=\{X_{1,g},X_{2,g},\cdots ,X_{NP,g}\},g=0,1,2,\cdots ,G\\ X_{i,g}=\{x_{i,g}^{1},x_{i,g}^{2},\cdots ,x_{i,g}^{D}\},i=1,2,\cdots ,NP\\ s.t.\;x_{i}^{min}\leq x_{i}^{j}\leq x_{i}^{max},j=1,2,\cdots ,D \end{array}\right.$$ (1)

where g and *G* denote the current and the maximum generation number. *NP* is the population size, *D* represents the dimension of the problem. $x_{i}^{min}$ and $x_{i}^{max}$ are the upper and lower boundaries of the solution space, respectively. The original population *P*_0_ is determined by random initiation in the solution space, and then the following cyclic evolution process is performed.

#### 2.1.2 Mutation

At generation g, a mutation individual $V_{i,g+1}=\{v_{i,g+1}^{1},v_{i,g+1}^{2},\cdots ,v_{i,g+1}^{D}\}$ is generated for each individual *X*_*i*,*g*_, commonly treated as follows.

$$V_{i,g+1}=X_{r1,g}+F∙(X_{r2,g}-X_{r3,g}),r1\neq r2\neq r3\neq i$$ (2)

where *X*_*r*1,g_, *X*_*r*2,g_ and *X*_*r*3,g_ are randomly selected individuals from the population *P*_g_, *r*1,*r*2,*r*3∈[1,2,⋯,*NP*]. The scaling factor *F* controls the amplification of the difference vector (*X*_*r*2,g_−*X*_*r*3,g_).

#### 2.1.3 Crossover

By means of binary crossover, the components are extracted from the target individual *X*_*i*,g_ and the mutation individual *V*_*i*,g+1_ to form the trial individual $U_{i,g+1}=\{u_{i,g+1}^{1},u_{i,g+1}^{2},\cdots ,u_{i,g+1}^{D}\}$.

$$u_{i,g+1}^{j}=\left\{ \begin{array}{l} v_{i,g+1}^{j},\;{rand}_{j}\leq CR\;or\;j=j_{rand}\\ x_{i,g}^{j},\;otherwise \end{array}\right.$$ (3)

where *rand*_*j*_ is a real random number in [0,1], *j*_*rand*_ is a random integer in [1,*D*]. The crossover probability *CR* determines the amount of replication from the mutation individual *V*_*i*,g+1_.

#### 2.1.4 Selection

After evaluating the fitness of the target individual and the trial individual, the winner goes on to the next generation.

$$X_{i,g+1}=\left\{ \begin{array}{c} U_{i,g+1},\;f(U_{i,g+1})\leq f(X_{i,g})\\ X_{i,g},\;otherwise \end{array}\right.$$ (4)

### 2.2 Gaining‑sharing knowledge algorithm

Gaining-sharing knowledge optimization algorithm (GSK) [11] is a nature-inspired algorithm that mimics the process of gaining and sharing knowledge throughout the human life, including the junior gaining-sharing phase and the senior gaining-sharing phase. In GSK, *D*_*junior*_ dimensions are randomly selected from each individual to adopt the junior scheme, and the remaining *D*_*senior*_ = *D*−*D*_*junior*_ dimensions to use the senior scheme. *D* is the dimension of the problem, and *D*_*junior*_ is determined by the following formula.

$$D_{junior}=D∙{\left(1-\frac{g}{G}\right)}^{k}$$ (5)

where the knowledge rate *k* is a constant, g and *G* represent the current and the maximum generation number.

#### 2.2.1 Junior gaining-sharing phase

In this phase, all individuals are arranged in ascending order according to fitness values: *X*_*best*,g_,⋯,*X*_*i*−1,g_,*X*_*i*,g_,*X*_*i*+1,g_⋯,*X*_*worst*,g_. When the Knowledge ratio *k*_*r*_>*rand*_*j*_ (a random number in [0,1]), the *j*th dimension of each individual remains unchanged. Otherwise, it is updated as follows.

$$x_{i,g+1}^{j}=\left\{ \begin{array}{l} x_{i,g}^{j}+k_{f}∙[\left(x_{i-1,g}^{j}-x_{i+1,g}^{j})+(x_{r,g}^{j}-x_{i,g}^{j}\right)],\;f(X_{i,g})>f(X_{r,g})\\ x_{i,g}^{j}+k_{f}∙[\left(x_{i-1,g}^{j}-x_{i+1,g}^{j})+(x_{i,g}^{j}-x_{r,g}^{j}\right)],\;f(X_{i,g})\leq f(X_{r,g}) \end{array}\right.$$ (6)

where the knowledge factor *k*_*f*_ is a real number greater than zero. $x_{i,g+1}^{j}$ and $x_{i,g}^{j}$ represent the *j*th dimension of *X*_*i*_ at the current generation and the next generation, respectively. $x_{i-1,g}^{j}$, $x_{i+1,g}^{j}$ and $x_{r,g}^{j}$ are the *j*th dimensional components of individuals *X*_*i*−1,g_, *X*_*i*+1,g_ and *X*_*r*,g_, respectively. *f*(*X*_*i*,g_) and *f*(*X*_*r*,g_) denote the fitness values of *X*_*i*,g_ and *X*_*r*,g_, respectively.

#### 2.2.2 Senior gaining-sharing phase

At this stage, after sorting by fitness values, all the individuals are divided into three groups: best people {*X*_*pb*,g_}, middle people {*X*_*m*,g_} and worst people {*X*_*pw*,g_}, with the number of 100*p*%, *N*−(2∙100*p*%), 100*p*%, respectively. Similarly, the *j*th dimension of each individual remains unchanged when *k*_*r*_>*rand*_*j*_, otherwise it is updated as follows.

$$x_{i,g+1}^{j}=\left\{ \begin{array}{l} x_{i,g}^{j}+k_{f}∙[\left(x_{rpb,g}^{j}-x_{rpw,g}^{j})+(x_{rm,g}^{j}-x_{i,g}^{j}\right)],f(X_{i,g})>f(X_{rm,g})\\ x_{i,g}^{j}+k_{f}∙[\left(x_{rpb,g}^{j}-x_{rpw,g}^{j})+(x_{i,g}^{j}-x_{rm,g}^{j}\right)],f(X_{i,g})\leq f(X_{rm,g}) \end{array}\right.$$ (7)

where $x_{rpb,g}^{j}$, $x_{rpw,g}^{j}$, $x_{rm,g}^{j}$ represent the *j*th dimension of individuals *X*_*rpb*,g_,*X*_*rpw*,g_,*X*_*rm*,g_, and individuals *X*_*rpb*,g_,*X*_*rpw*,g_,*X*_*rm*,g_ are randomly selected from groups {*X*_*pb*,g_}, {*X*_*pw*,g_}, {*X*_*m*,g_}.

### 2.3 Harris hawks optimization

Harris hawks optimization (HHO) is a novel swarm-based algorithm proposed by Heidari et al. [10], which imitates the cooperative behavior and chase pattern of Harris hawks in the process of hunting. In HHO, there are three primary phases: exploration, transition from exploration to exploitation, exploitation.

#### 2.3.1 Exploration phase

At this phase, the hawks use the following two strategies to find prey.

$$X_{i,g+1}=\left\{ \begin{array}{c} X_{rand,g}-r_{1}∙|X_{rand,g}-2∙r_{2}∙X_{i,g}|,\;q\geq 0.5\\ (X_{rabbit,g}-X_{mean,g})-r_{3}∙(LB+r_{4}∙(UB-LB)),\;q<0.5 \end{array}\right.$$ (8)

$$X_{mean,g}=\frac{1}{N}\sum_{i=1}^{N}X_{i,g}$$ (9)

where *X*_*mean*,g_ and *X*_*i*,g_ denote the mean and current location vector of the Harris hawk at the current generation g, *X*_*rand*,g_ and *X*_*rabbit*,g_ are positions of a randomly selected hawk and the prey. *X*_*i*,g+1_ indicates the location vector of the hawk at the next generation g+1. *r*_1_, *r*_2_, *r*_3_, *r*_4_ and *q* are real random numbers in [0,1], *UB* and *LB* are the upper and lower range, respectively.

#### 2.3.2 Transition from exploration to exploitation

Through the rabbit’s escaping energy *E*, the HHO algorithm can realize the transition from exploration to exploitation. The escaping energy *E* is formulated as:

$$E=2∙E_{0}∙\left(1-\frac{g}{G}\right)$$ (10)

where g and *G* indicate the current and the maximum generation number, *E*_0_ is the initial energy in (−1,1).

#### 2.3.3 Exploitation phase

According to the escaping energy *E* and the successful escaping chance *r* of the prey, diverse exploitative behaviors are adopted, such as soft besiege, hard besiege, soft besiege with progressive rapid dives and hard besiege with progressive rapid dives. The successful escaping chance *r* is a real random number in [0,1].

- **Soft besiege** (*r*≥0.5 and |*E*|≥0.5). The Harris hawks softly encircled the prey, modelled as follows.

$$X_{i,g+1}=\Delta X_{i,g}-E∙|J∙X_{rabbit,g}-X_{i,g}|$$ (11)

$$\Delta X_{i,g}=X_{rabbit,g}-X_{i,g}$$ (12)

where *J* = 2∙(1−*r*_5_) indicates the random jump intensity of the prey, and *r*_5_ is a real random number in [0,1].

- **Hard besiege** (*r*≥0.5 and |*E*|≥0.5). The Harris hawks hardly encircled the prey, and their positions are updated as follows:

$$X_{i,g+1}=X_{rabbit,g}-E∙|\Delta X_{i,g}|$$ (13)

where Δ*X*_*i*,g_ is the difference between positions of the rabbit and the current hawk, which can be seen in Eq (12).

- **Soft besiege with progressive rapid dives** (*r*<0.5 and |*E*|≥0.5). The prey still has enough energy to escape, and the Harris hawks respond as follows.

$$Y=X_{rabbit,g}-E∙|J∙X_{rabbit,g}-X_{i,g}|$$ (14)

Z=Y+S∙LF(D) (15)

$$X_{i,g+1}=\left\{ \begin{array}{l} Y,\;if\;f(Y)<f(X_{i,g})\\ Z,\;if\;f(Z)<f(X_{i,g}) \end{array}\right.$$ (16)

where *f*(*Y*) and *f*(*Z*) represent the fitness values of *Y* and *Z*, respectively. *D* denotes the dimension of the problem, *LF*(*D*) is the Levy fight that can be obtained through the following formula.

$$LF(D)=0.01∙\frac{u∙\sigma }{{\left|v\right|}^{1/\beta }},\sigma ={\left(\frac{\Gamma (1+\beta )∙\sin\left(\pi \beta /2\right)}{\Gamma (1+\beta /2)∙\beta ∙2^{\left(\beta -1/2\right)}}\right)}^{1/\beta }$$ (17)

where *u* and *v* are random numbers in [0,1], *β* is a constant value of 1.5.

- **Hard besiege with progressive rapid dives** (*r*<0.5 and |*E*|≥0.5). In contrast to the previous behavior, the rabbit’s escaping energy is insufficient, and the behavior of the Harris hawks are modelled as follows.

$$Y=X_{rabbit,g}-E∙|J∙X_{rabbit,g}-X_{mean,g}|$$ (18)

Z=Y+S∙LF(D) (19)

$$X_{i,g+1}=\left\{ \begin{array}{l} Y,\;if\;f(Y)<f(X_{i,g})\\ Z,\;if\;f(Z)<f(X_{i,g}) \end{array}\right.$$ (20)

where *X*_*mean*,g_ is the average position calculated by Eq (9).

## 3 The proposed algorithm

This section is a detailed introduction to the proposed algorithm, including its motivation, hybrid mutation operator and Crossover probability self-adaption.

### 3.1 Motivations

According to the above introduction, changes based on DE components and hybridization with other meta-heuristic algorithms can improve the performance of DE. As for GSK algorithm, its two-stage model has been able to balance exploration and exploitation effectively [11]. On this basis, a mutation strategy “DE/rand/1” with global exploration ability and HHO’s Soft Obsessed strategy with exploitation ability are considered. By applying these four strategies to mutation operation, a balanced double insurance mechanism for exploration and exploitation is formed.

Besides, for most DE variants, the operations of mutation, crossover and selection are relatively independent. In DEGH, these operations are linked together by the control parameters *F*, *CR* and a binary variable *h* that records the historical evolution state, making the connection within the whole DE framework even tighter.

### 3.2 Hybrid mutation operator

In order to achieve a better balance between exploration and exploitation, DEGH adopts a dual insurance mechanism in the mutation operation, which contains four mutation strategies. First, the strategy of GSK in the junior phase (Eq (6) and senor phase (Eq 7) in GSK are introduced and streamlined, which help maintain a sufficient balance between global exploration and local exploitation capabilities in the search process [44]. The two strategies are as abbreviated as GSK/J-mutation and GSK/S-mutation. Second, in order to further strengthen this balance, DE’s classic mutation strategy "DE/rand/1" and the soft besiege in the exploitation phase of HHO are added to the hybrid mutation operator, which are called “DE/rand /1-mutation” and “HHO/SB-mutation”, respectively. Thus, GSK/J-mutation and GSK/S-mutation, combined with DE/rand/1-mutation and HHO/SB-mutation, form a hybrid mutation operator, which is a dual-insurance mechanism for balancing global exploration and local exploitation capabilities.

**Fig 1:**
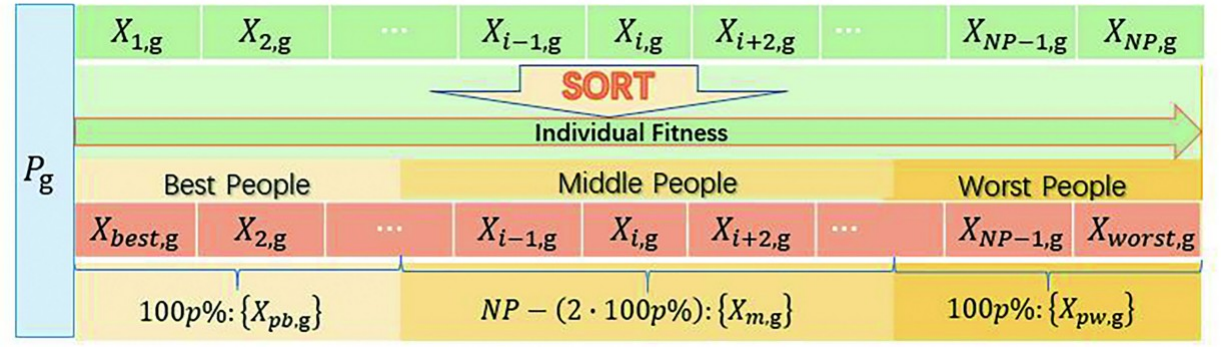
Schematic of population sequencing and grouping in DEGH.

Before mutation operation, all individuals are arranged according to fitness values to form a new population *P*_*g*_ = {*X*_*best*,*g*_,*X*_2,*g*_,⋯,*X*_*NP*−1,*g*_,*X*_*worst*,*g*_}, which is grouped into best people {*X*_*pb*,g_}, middle people {*X*_*pw*,g_} and worst people {*X*_*m*,g_}, as shown in Fig 1. The population sequencing and grouping strategy of DEGH is the same as that of GSK. On this basis, two random distribution numbers *R*1_*i*,g_ and *R*2_*i*,g_, as well as control parameters *F* and *CR*_*i*,*g*_, together determine the mutation strategy adopted by each individual. Among them, *R*1_*i*,g_,*R*2_*i*,g_ and *CR*_*i*,*g*_ are implemented at the individual level.

#### 3.2.1 GSK/J-mutation

When *R*1_*i*,g_≥*F* and *R*2_*i*,g_<*CR*_*i*,*g*_, the strategy of the junior phase (Eq (6)) of GSK is improved. The scaling factor *F* is substituted for the knowledge factor *k*_*f*_, and the mutation individual *V*_*i*,g+1_ generated is as follows.

$$V_{i,g+1}=X_{i,g}+F∙(X_{i-1,g}-X_{i+1,g})+F∙(X_{r,g}-X_{i,g})$$ (21)

where *X*_*i*−1,g_ and *X*_*i*+1,g_ are the nearest better and worsen individuals of the target individual *X*_*i*,g_. if *X*_*i*,g_ is *X*_*best*,g_, *X*_*i*−1,g_ and *X*_*i*+1,g_ are *X*_2,g_ and *X*_3,g_. if *X*_*i*,g_ is *X*_*worst*,g_, *X*_*i*−1,g_ and *X*_*i*+1,g_ are *X*_*NP*−2,g_ and *X*_*NP*−1,g_. *X*_*r*,g_ denotes a randomly selected individual in the new population *P*^*G*^.

#### 3.2.2 GSK/S-mutation

When *R*1_*i*,g_<*F* and *R*2_*i*,g_≥*Cr*_*i*,g_, similarly, the strategy in Eq (7) of the senior phase of GSK is also changed, and the mutation individual *V*_*i*,g+1_ is generated by the following mode.

$$V_{i,g+1}=X_{i,g}+F∙(X_{rpb,g}-X_{rpw,g})+F∙(X_{i,g}-X_{rm,g})$$ (22)

where *X*_*rpb*,g_, *X*_*rpw*,g_ and *X*_*rm*,g_ are randomly chosen individuals from best people {*X*_*pb*,g_}, middle people {*X*_*pw*,g_} and worst people {*X*_*m*,g_}, respectively.

#### 3.2.3. DE/rand/1-mutation

when *R*1_*i*,g_≥*F* and *R*2_*i*,g_≥*CR*_*i*,*g*_, the mutation individual *V*_*i*,g+1_ is produced by the classic mutation operator of DE in Eq (2), which is famous for its strong global search capability.

#### 3.2.4 HHO/SB-mutation

when *R*1_*i*,g_<*F* and *R*2_*i*,g_<*CR*_*i*,g_, according to the enhanced version of the soft besiege rule of exploitation phase in HHO, the mutation individual *V*_*i*,g+1_ is obtained as follows.

$$V_{i,g+1}=\Delta X+F∙(X_{best,g}-X_{i,g})$$ (23)

$$\Delta X=X_{best,g}-X_{i,g}$$ (24)

### 3.3 Crossover probability self-adaption

As shown in the mutation operation above, the crossover probability *CR* affects the selection of the mutation operator adopted by each individual. In order to make the internal phases of DE more closely linked, the adjustment of *CR* is associated with mutation and selection operations.

At each generation of DEGH, the frequencies used for GSK/J-mutation, GSK/S-mutation, DE/rand/1-mutation and HHO/SB-mutation are counted and represented as *anum*,*bnum*, *cnum* and *dnum* respectively. At the same time, the mutation strategy adopted by each individual is labelled with *flag*: individuals with GSK/J-mutation are *flag* = 1; individuals with GSK/S-mutation are *flag* = 2; individuals with DE/rand/1-mutation are *flag* = 3; individuals with HHO/SB-mutation are *flag* = 4. Besides, in the selection operation of DEGH, a binary variable *h* recording the evolutionary status of the trial individual is introduced and participated in the adjustment of CR. If the trial individual fails to evolve, *h*_*i*,*g*+1_ is set to 0 and CR is assigned a random number in [0,1]. On the contrary, *h*_*i*,*g*+1_ is set to 1 and the adaptive adjustment of *CR* is as follows.

$$CR_{i}=\{\begin{array}{l} \mathrm{anum}/NP\;,\;\mathrm{if}\;{\mathrm{flag}}_{i}=1\\ \mathrm{bnum}/NP\;,\;\mathrm{if}\;{\mathrm{flag}}_{i}=2\\ \mathrm{cnum}/NP\;,\;\mathrm{if}\;{\mathrm{flag}}_{i}=3\\ \mathrm{dnum}/NP\;,\;\mathrm{if}\;{\mathrm{flag}}_{i}=4 \end{array}$$ (25)

where *flag*_*i*_ records the mutation strategy applied by individual *X*_*i*,g_ and *NP* is the population size.

### 3.4 Pseudocode of the proposed algorithm

**Fig 2:**
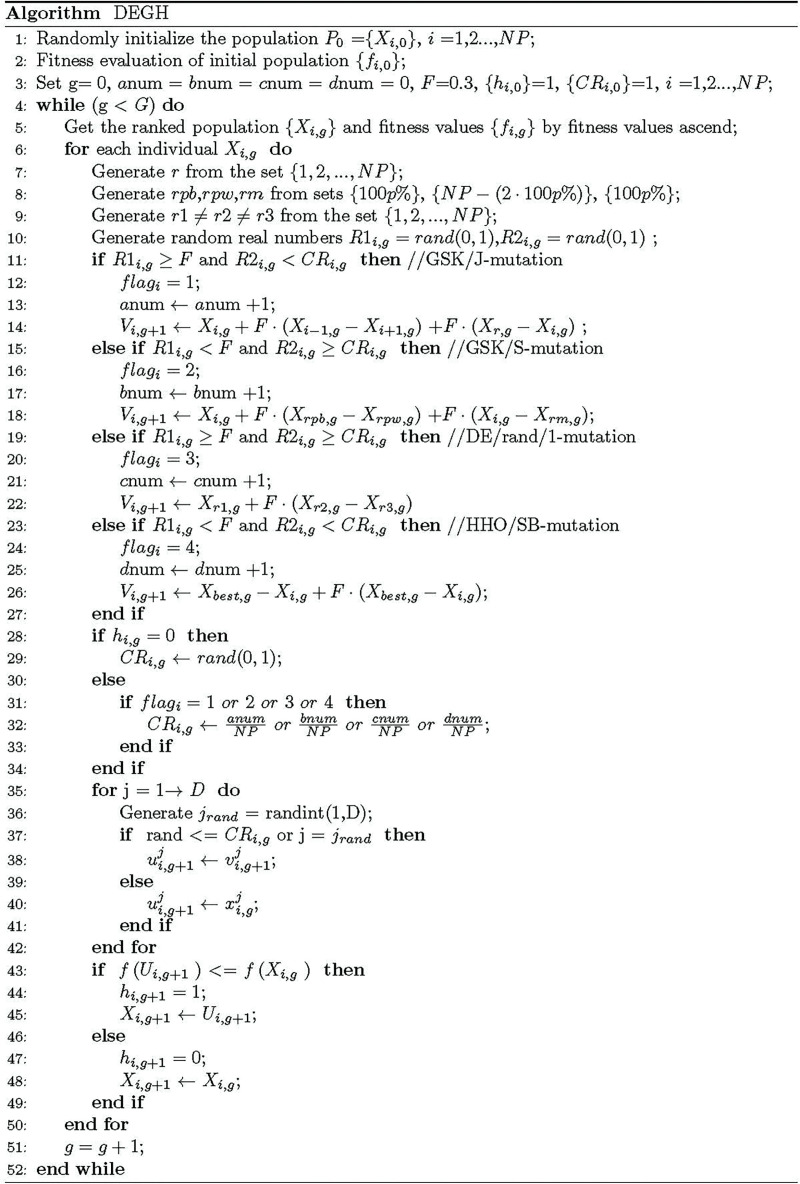
The pseudo-code of DEGH.

Based on the above description, pseudo-code of the proposed DEGH algorithm is reported in Fig 2, where the hybrid mutation operator is shown in lines 11–27 and the crossover probability self-adaptation strategy is used in lines 28–34.

### 3.5 Computational complexity

The computational complexity of the DEGH depends on the following aspects: initialization, sorting, evaluation, mutation, crossover, and selection. Compared with the original DE, DEGH only increases the complexity of sorting. The computational complexity of the initial DE is *O*(*NP*∙*D*∙*G*), and the sorting complexity is *O*(*NP*), so in general, the computational complexity of DEGH remains the same as the original DE, which is *O*(*NP*∙*D*∙*G*).

## 4 Experimental results and analysis

The performance of the proposed DEGH is evaluated by 32 well-known benchmark functions [45, 46] listed in Table 1, in which *f*_1_~*f*_14_ are unimodal functions and *f*_15_~*f*_32_ are multimodal functions. Besides, DEGH is compared with eight enhanced DE algorithms including IMMSADE [19], CIPDE [20], EBDE [21], EDE [21], EJADE [30], LSHADE-SPACMA [35], DEPSO [37] and ATLDE [39] at *D* = 30,100. The former five algorithms are based on changes in DE components, while the latter three algorithms are mixtures of DE and other meta-heuristic algorithms.

**Table 1:** The benchmark test functions.

Name	Function	Domain	*f*(*)
Sphere	$f_{1}(x)=\sum_{i=1}^{D}{x_{i}}^{2}$	[−100,100]^*D*^	0
Elliptic	$f_{2}(x)=\sum_{i=1}^{D}{\left(10^{6}\right)}^{\frac{i-1}{D-1}}{x_{i}}^{2}$	[−100,100]^*D*^	0
Bent Cigar	$f_{3}(x)={x_{1}}^{2}+10^{6}\sum_{i=2}^{D}{x_{i}}^{2}$	[−100,100]^*D*^	0
Schwefel 1.2	$f_{4}(x)=\sum_{i=1}^{D}{\left(\sum_{j=1}^{i}x_{j}\right)}^{2}$	[−100,100]^*D*^	0
Schwefel 2.22	$f_{5}(x)=\sum_{i=1}^{D}\left|x_{i}\right|+\prod_{i=1}^{D}\left|x_{i}\right|$	[−10,10]^*D*^	0
Schwefel 2.21	*f*_6_(x) = *max*{|*x*_*i*_|, 1≤*i*≤*D*}	[−100,100]^*D*^	0
Sum of Different Power	$f_{7}(x)=\sum_{i=1}^{D}{\left|x_{i}\right|}^{i+1}$	[−100,100]^*D*^	0
Sum Squares	$f_{8}(x)=\sum_{i=1}^{D}i{x_{i}}^{2}$	[−10,10]^*D*^	0
Discus	$f_{9}(x)=10^{6}{x_{1}}^{2}+\sum_{i=2}^{D}{x_{i}}^{2}$	[−100,100]^*D*^	0
Different Powers	$f_{10}(x)=\sqrt{\sum_{i=1}^{D}{\left|x_{i}\right|}^{2+4\frac{i-1}{D-1}}}$	[−100,100]^*D*^	0
Exponential	$f_{11}(x)=-exp(-0.5\sum_{i=1}^{D}{x_{i}}^{2})$	[−1,1]^*D*^	-1
Zakharov	$f_{12}(x)=\sum_{i=1}^{D}{x_{i}}^{2}+{\left(\sum_{i=1}^{D}0.5x_{i}\right)}^{2}+{\left(\sum_{i=1}^{D}0.5x_{i}\right)}^{4}$	[−5,10]^*D*^	0
Step	$f_{13}(x)=\sum_{i=1}^{D}{\left(\left|x_{i}+0.5\right|\right)}^{2}$	[−100,100]^*D*^	0
Noise quartic	$f_{14}(x)=\sum_{i=1}^{D}i{x_{i}}^{4}+rand[0,1)$	[−1.28,1.28]^*D*^	0
Rosenbrock	$f_{15}(x)=\sum_{i=1}^{D-1}\left[100{∙({x_{i}}^{2}-x_{i+1})}^{2}+{\left(x_{i}-1\right)}^{2}\right]$	[−30,30]^*D*^	0
Griewank	$f_{16}(x)=\sum_{i=1}^{D}{x_{i}}^{2}/4000-\prod_{i=1}^{D}\cos\left(x_{i}/\sqrt{i}\right)+1$	[−600,600]^*D*^	0
Rastrigin	$f_{17}(x)=\sum_{i=1}^{D}\left({x_{i}}^{2}-10\;\cos\left(2\pi x_{i}\right)+10\right)$	[−5.12,5.12]^*D*^	0
Apline	$f_{18}(x)=\sum_{i=1}^{D}\left|x_{i}\;\sin\;x_{i}+0.1x_{i}\right|$	[−100,100]^*D*^	0
Bohachevsky_2	$f_{19}(x)=\sum_{i=1}^{D-1}\left[{x_{i}}^{2}+{2x}_{i+1}^{2}-0.3\;\cos\left(3\pi x_{i}\right)\cos\left(3\pi x_{i+1}\right)+0.3\right]$	[−100,100]^*D*^	0
Salomon	$f_{20}(x)=1-\cos\left(2\pi \sqrt{\sum_{i=1}^{D}{x_{i}}^{2}}\right)+0.1\sqrt{\sum_{i=1}^{D}{x_{i}}^{2}}$	[−100,100]^*D*^	0
Scaffer2	$f_{21}(x)=\sum_{i=1}^{D}{\left({x_{i}}^{2}+x_{i+1}^{2}\right)}^{0.25}(\sin\left(50{\left(x_{i}^{2}+x_{i+1}^{2}\right)}^{0.1}\right)+1)\;x_{D+1}=x_{1}$	[−100,100]^*D*^	0
Ackley	$f_{22}(x)=-20exp(-0.2\sqrt{\sum_{i=1}^{D}{x_{i}}^{2}/D})-exp(\sum_{i=1}^{D}\cos\left(2\pi x_{i}\right)/D)+20+e$	[−32,32]^*D*^	0
Weierstrass	$f_{23}(x)=\sum_{i=1}^{D}\left(\sum_{k=0}^{k_{max}}\left[a^{k}\;\cos\left(2\pi b^{k}(x_{i}+0.5)\right)\right]\right)-D\sum_{k=0}^{k_{max}}\left[a^{k}\cos\left(2\pi b^{k}∙0.5\right)\right]$	[−0.5,0.5]^*D*^	0
	*a* = 0.5, *b* = 3, *k*_*max*_ = 20		
Katsuura	$f_{24}(x)=\frac{10}{D^{2}}\prod_{i=1}^{D}{\left(1+i\sum_{j=1}^{32}\frac{\left|2^{j}x_{i}-round(2^{j}x_{i})\right|}{2^{j}}\right)}^{\frac{10}{D^{1.2}}}-\frac{10}{D^{2}}$	[−100,100]^*D*^	0
HappyCat	$f_{25}(x)={\left|\sum_{i=1}^{D}{x_{i}}^{2}-D\right|}^{1/4}+\left(0.5\sum_{i=1}^{D}{x_{i}}^{2}+\sum_{i=1}^{D}x_{i}\right)/D+0.5$	[−100,100]^*D*^	0
HGBat	$f_{26}(x)={\left|{\left(\sum_{i=1}^{D}{x_{i}}^{2}\right)}^{2}-{\left(\sum_{i=1}^{D}x_{i}\right)}^{2}\right|}^{1/2}+\left(0.5\sum_{i=1}^{D}{x_{i}}^{2}+\sum_{i=1}^{D}x_{i}\right)/D+0.5$	[−100,100]^*D*^	0
Scaffer’s F6	$f_{27}(x)=\sum_{i=1}^{D}\left(0.5+\left({\left(\sin\left(\sqrt{{x_{i}}^{2}+x_{i+1}^{2}}\right)\right)}^{2}-0.5\right)/{\left(1+0.001({x_{i}}^{2}+x_{i+1}^{2})\right)}^{2}\right)\;x_{D+1}=x_{1}$	[−0.5,0.5]^*D*^	0
Expanded Scaffer	*f*_28_(x) = *f*_27_(*x*_1_, *x*_2_)+*f*_27_(*x*_2_,*x*_3_)+⋯+*f*_27_(*x*_*D*−1_, *x*_*D*_)+*f*_27_(*x*_*D*_, *x*_1_)	[−5,5]^*D*^	0
Griewank+Rosenbrock	*f*_29_(*x*) = *f*_16_(*f*_15_(*x*_1_,*x*_2_))+*f*_16_(*f*_15_(*x*_2_, *x*_3_))+⋯+*f*_16_(*f*_15_(*x*_*D*−1_,*x*_*D*_))+*f*_16_(*f*_15_(*x*_*D*_, *x*_1_))	[−5.12,5.12]^*D*^	0
NCRastrigin	$f_{30}(x)=\sum_{i=1}^{D}\left[{y_{i}}^{2}-10\;\cos\left(2\pi y_{i}\right)+10\right],y_{i}=\left\{ \begin{array}{c} x_{i},|x_{i}|<0.5\\ \frac{round(2x_{i})}{2},|x_{i}|\geq 0.5 \end{array}\right.$	[−10,10]^*D*^	0
Levy and Montalvo 1	$f_{31}(x)=\frac{\pi }{D}\{10{\left(\sin\left(\pi y_{1}\right)\right)}^{2}+\sum_{i=1}^{D-1}{\left(y_{i}-1\right)}^{2}[1+10{\left(\sin\left(\pi y_{i+1}\right)\right)}^{2}]+{\left(y_{D}-1\right)}^{2}\}+\sum_{i=1}^{D}u(x_{i},10,100,4)$	[−10,10]^*D*^	0
	$y=1+\frac{1}{4}(x_{i}+1),u(x_{i},a,k,m)=\left\{ \begin{array}{l} k{\left(x_{i}-a\right)}^{m}\;,\;x_{i}>a\\ 0\;,-a\leq x_{i}\leq a\\ k{\left({-x}_{i}-a\right)}^{m}\;,x_{i}<-a \end{array}\right.$		
Levy and Montalvo 2	$f_{32}(x)=0.1\{10{\left(\sin\left(3\pi x_{1}\right)\right)}^{2}+\sum_{i=1}^{D-1}{\left(x_{i}-1\right)}^{2}[1+{\left(\sin\left(3\pi x_{i+1}\right)\right)}^{2}]+{\left(x_{D}-1\right)}^{2}[1+{\left(\sin\left(2\pi x_{D}\right)\right)}^{2}]\}+\sum_{i=1}^{D}u(x_{i},5,100,4)$	[−5,5]^*D*^	0

### 4.1 Experimental setting

In the following experiments, to ensure a fair comparison, the common parameters of all algorithms are set the same: the maximum generation number G is set to 1000, the population size NP is set to 100, and 30 independent runs are conducted. Other parameter settings of each algorithm are shown in Table 2.

**Table 2:** The parameter settings of compared algorithms.

Algorithm	Parameters
IMMSADE	*τ* = 0.7, *λ*∈[0.7,1.0], *F*∈[0.1,0.8], *CR*∈[0.3,10]
CIPDE	*c* = 0.1, *μ*_*F*_ = 0.7, *μ*_*CR*_ = 0.5
EBDE	*p* = 0.1, H = 100, *M*_*F*_(1:H) = *M*_*CR*_(1:H) = 0.5, *F* = *randn*(*M*_*F*_, 0.1), *CR* =*randn*(*M*_*CR*_, 0.1)
EDE	H = 100, *M*_*F*_(1:H) = *M*_*CR*_(1:H) = 0.5, *F* = *randn*(*M*_*F*_, 0.1), *CR* =*randn*(*M*_*CR*_, 0.1)
EJADE	*μ*_*F*_ = *μ*_*CR*_ = 0.5, *c* = 0.1, *p* = 0.05, *F* = *randn*(*μ*_*F*_, 0.1), *CR* = *randn*(*μ*_*CR*_, 0.1)
LSHADE-SPACMA	*Pbest* = 0.11, H = 1.4, *Arc_rate* = 5, *F*_*CP*_ =s 0.5, *c* = 0.8
DEPSO	*c*_1_ = *c*_2_ = 2, *ω*∈[0.4,0.9], *CR*∈[0.3,1.0], *F*∈[0.1,0.8], *NS*_*max*_ = 5, *γ* = 0.001, *τ* = 0.7, *SEP* = 0.4*∙NP*
ATLDE	*CR* = 0.9, *ε* = 0.5
DEGH	*F* = 0.3

### 4.2 Parameter study

In this section, the sensitivity analysis of population size NP and scaling factor and the efficiency analysis of crossover probability are studied through relevant experiments.

#### 4.2.1 Sensitivity analysis to population size

**Fig 3:**
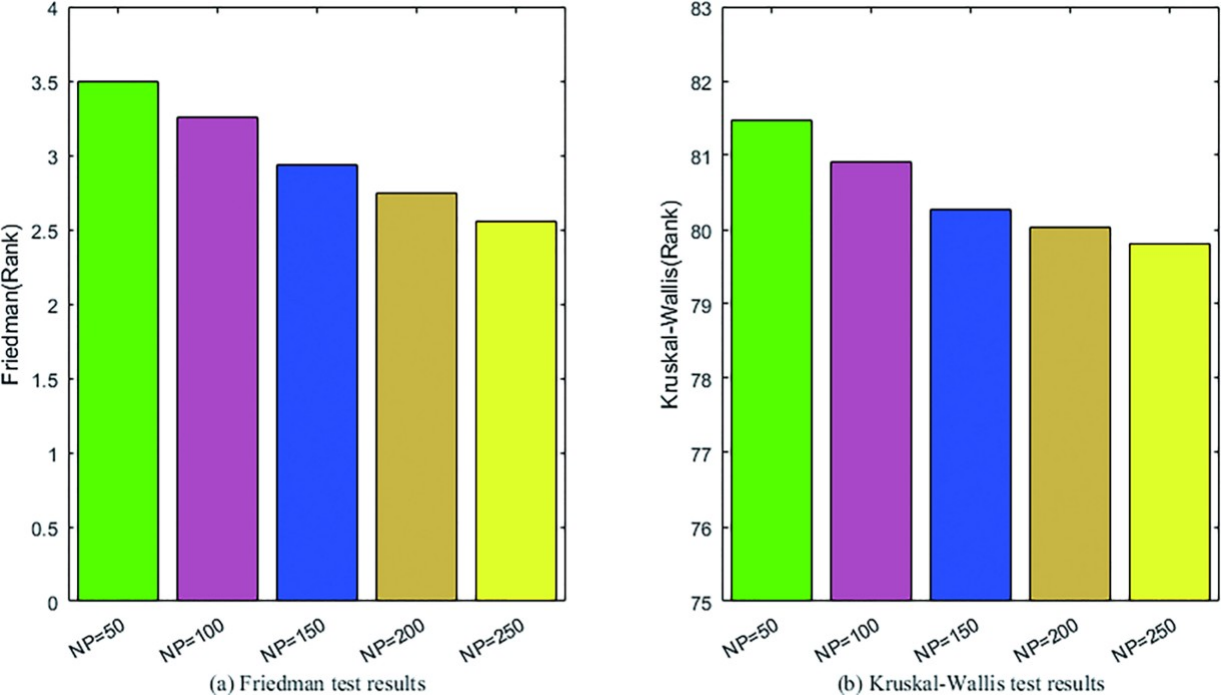
The results of Friedman and Kruskal-Wallis tests for DEGHs with different population size.

As one of the control parameters of DE, the influence of population size NP on the performance of DEGH is studied on the 32 benchmark functions at *D* = 30. DEGH variants with *NP* = 50,150,200,250 are compared with the standard DEGH with *NP* = 100, the optimization results of which are evaluated by Friedman, Kruskal-Wallis and Wilcoxon’s rank-sum tests [47]. The statistical tests results are shown in Fig 3 and Table 3.

**Table 3:** The results of Wilcoxon’s rank-sum test between DEGH with NP = 250 and DEGHs with different population size.

DEGH with *NP* = 250 vs.	*R*^+^	*R*^−^	*p*−*value*	*α* = 0.05	*α* = 0.1
DEGH with *NP* = 50	36	0	8.95e-01	No	No
DEGH with *NP* = 100	36	0	9.09e-01	No	No
DEGH with *NP* = 150	32	4	9.51e-01	No	No
DEGH with *NP* = 200	32	4	9.65e-01	No	No

“No” represents no significant performance discrepancy between the two compared.

As can be seen from Fig 3, with the increase of *NP*, the performance of DEGH improves. DEGH performs best at *NP* = 250. From the data listed in Table 3, there is no significant difference in the performance of DEGHs with different NP values, that is, DEGH is not sensitive to population size *NP*. In order not to lose universality, the population size *NP* is set to 100 in the following experiments.

#### 4.2.2 Sensitivity analysis to scaling factor

**Fig 4:**
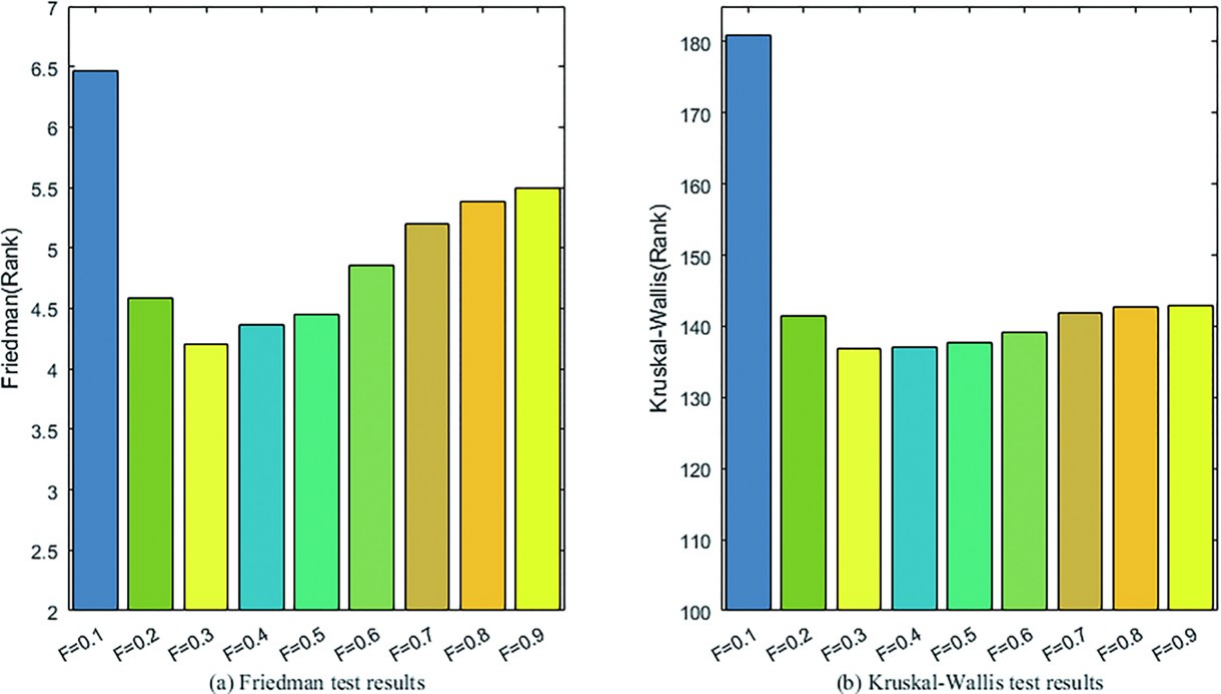
The results of the Friedman and Kruskal-Wallis tests for DEGHs with different *F* values.

In DEGH, the scaling factor *F* plays a vital role in the mutation operation. By setting *F* to *F*∈[0.1,0.9] in steps of 0.1, a series of experiments are conducted to analyze the sensitivity of the scaling factor. Three nonparametric statistical tests are used to analyze the optimization results of 30-dimensional problems with different *F* values, which are recorded in Fig 4 and Table 4, respectively.

**Table 4:** The results of Wilcoxon’s rank-sum test between DEGH with *F* = 0.3 and DEGHs with different *F* values.

DEGH with *F* = 0.3 vs.	*R*^+^	*R*^−^	*p*−*value*	*α* = 0.50	*α* = 0.1
DEGH with *F* = 0.1	173	37	1.43e-02	Yes	Yes
DEGH with *F* = 0.2	24	21	7.49e-01	No	No
DEGH with *F* = 0.4	22	14	9.51e-01	No	No
DEGH with *F* = 0.5	24	12	9.37e-01	No	No
DEGH with *F* = 0.6	31	5	9.09e-01	No	No
DEGH with *F* = 0.7	35	1	8.12e-01	No	No
DEGH with *F* = 0.8	35	1	7.84e-01	No	No
DEGH with *F* = 0.9	35	1	7.84e-01	No	No

“Yes” indicates DEGH with *F* = 0.3 outperforms other DEGHs with different *F* values. “No” represents no significant performance discrepancy between the two compared. The bigger the *R*^+^, the better the first algorithm.

From Fig 4, it is clear that the performance of DEGH is best at *F* = 0.3. From Table 2, it can be seen that DEGH is insensitive to *F* except *F* = 0.1. Therefore, F = 0.3 can be considered as a suitable value for subsequent experiments.

#### 4.2.3 Efficiency analysis to crossover probability

**Fig 5:**
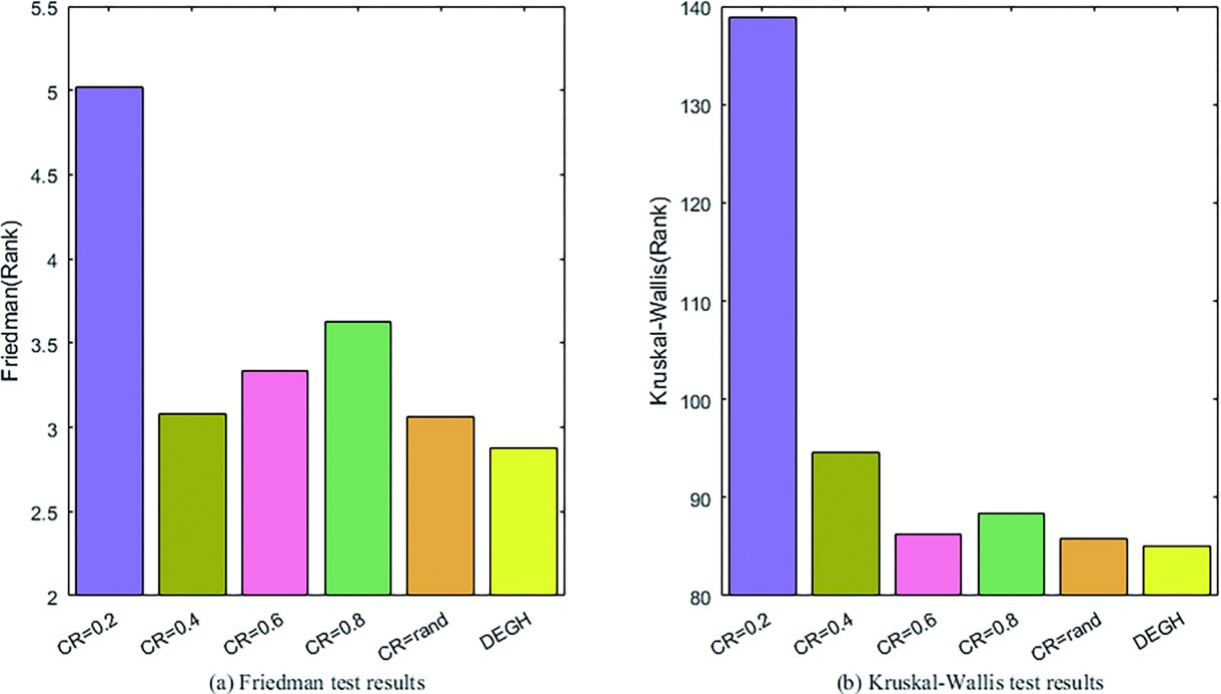
The results of Friedman and Kruskal-Wallis tests for DEGH and its variants with different *CR* values.

In order to investigate the effectiveness of crossover probability self-adaptation strategy in DEGH, the efficiency of crossover probability is analyzed by setting *CR* = 0.2,0.4,0.8, *rand* and compared with the proposed DEGH, where *rand* represents a random real number inside [0,1]. The results of the non-parametric statistical tests of these DEGHs are shown in Fig 5 and Table 5.

**Table 5:** The results of Wilcoxon’s rank-sum test between DEGH and its variants with different *F* values.

DEGH vs.	*R*^+^	*R*^−^	*p*−*value*	*α* = 0.05	*α* = 0.1
DEGH with *CR* = 0.2	357	21	2.13e-05	Yes	Yes
DEGH with *CR* = 0.4	47	19	4.40e-01	No	No
DEGH with *CR* = 0.6	32	4	9.23e-01	No	No
DEGH with *CR* = 0.8	34	2	8.81e-01	No	No
DEGH with *CR* = *rand*	20	16	9.51e-01	No	No

“Yes” represents that the combined DEGH’s performance is better than its variant significantly. “No” represents no significant performance discrepancy between the two compared. The bigger the *R*^+^, the better the combined DEGH.

From Fig 5, it is evident that the proposed DEGH is the best and DEGH with *CR* = *rand* is the second best. It can be concluded from Table 5 that, except for DEGH with *CR* = 0.2, there is no significant performance difference between DEGH and its variants. In other words, the crossover probability self-adaption is effective, but DEGH is less susceptible to crossover probability.

### 4.3 Comparison with eight state-of-the-art DE variants

In order to comprehensively evaluate the performance of the proposed algorithm, the optimization results and convergence properties of DEGH and eight enhanced DE algorithms on 32 benchmark functions at *D* = 30,50,100 are compared and analyzed.

#### 4.3.1 Optimization results

The optimization results of each algorithm at *D* = 30, *D* = 50 and *D* = 100 are listed in Tables 6–8 respectively, where Mean and STD refer to the average and standard deviation of the function error value over 30 independent runs. Besides, the Wilcoxon signed-rank test results on each dimensional problem are shown in Table 9, where the symbol “+/−/≈” indicates the performance of DEGH is “better than/worse than/similar to” the compared algorithm.

**Table 6:** Mean and STD obtained by eight enhanced DEs and DEGH on benchmark functions at 30D.

F	IMMSADE	CIPDE	EBDE	EDE	EJADE	LSHADE-SPACMA	DEPSO	ATLDE	DEGH
	Mean ± STD	Mean ± STD	Mean ± STD	Mean ± STD	Mean ± STD	Mean ± STD	Mean ± STD	Mean ± STD	Mean ± STD
***f***_**1**_	2.21E-29±9.12E-29	2.91E-43±1.19E-42	1.40E-48±5.00E-48	2.32E-38±3.98E-38	2.10E-39±3.34E-39	1.60E-63±2.74E-63	3.35E-93±1.60E-92	1.50E-54±2.50E-54	**0.00E+00±0.00E+00**
***f***_**2**_	1.58E-23±8.61E-23	3.62E-40±1.16E-39	2.27E-43±6.50E-43	1.71E-33±5.63E-33	1.06E-34±3.92E-34	1.83E-54±3.29E-54	6.00E-96±2.26E-95	1.41E-50±1.92E-50	**0.00E+00±0.00E+00**
***f***_**3**_	8.11E-22±3.43E-21	3.52E-38±1.09E-37	3.99E-41±1.45E-40	7.84E-31±2.25E-30	1.02E-31±4.32E-31	1.07E-51±4.73E-51	7.32E-93±4.01E-92	4.17E-48±1.34E-47	**0.00E+00±0.00E+00**
***f***_**4**_	3.07E+00±7.02E+00	2.35E-10±8.88E-10	1.07E-12±2.78E-12	5.94E-05±1.08E-04	8.10E-10±1.37E-09	4.24E-31±1.55E-30	4.49E-91±2.46E-90	5.68E-53±2.09E-52	**0.00E+00±0.00E+00**
***f***_**5**_	8.87E-16±2.95E-15	2.27E-20±7.54E-20	6.77E-26±1.51E-25	5.55E-21±9.88E-21	6.79E-17±1.63E-16	1.56E-33±1.39E-33	3.89E-50±2.08E-49	2.05E-26±3.84E-26	**0.00E+00±0.00E+00**
***f***_**6**_	3.55E-08±6.88E-08	1.45E-08±3.89E-08	7.09E-08±9.93E-08	6.29E-03±7.53E-03	2.01E-04±2.46E-04	7.05E-23±1.66E-22	1.73E-49±8.89E-49	5.83E-23±7.42E-23	**0.00E+00±0.00E+00**
***f***_**7**_	1.33E-83±7.22E-83	3.18E-55±1.74E-54	2.86E-64±1.57E-63	1.86E-35±7.83E-35	5.59E-71±2.13E-70	2.61E-69±1.36E-68	3.44E-113±1.87E-112	2.68E-115±1.06E-114	**0.00E+00±0.00E+00**
***f***_**8**_	3.29E-26±1.78E-25	5.84E-45±2.33E-44	4.81E-50±9.75E-50	6.11E-39±7.36E-39	3.06E-40±5.20E-40	8.50E-68±1.23E-67	2.81E-99±1.53E-98	1.03E-54±3.32E-54	**0.00E+00±0.00E+00**
***f***_**9**_	1.86E-27±5.96E-27	1.08E-44±2.56E-44	9.04E-48±2.00E-47	1.66E-36±2.78E-36	4.08E-37±1.79E-36	9.90E-58±4.49E-57	1.13E-99±4.45E-99	1.04E-53±2.10E-53	**0.00E+00±0.00E+00**
***f***_**10**_	1.34E-20±7.29E-20	1.79E-27±2.90E-27	3.08E-25±9.17E-25	1.88E-16±4.26E-16	4.33E-25±5.22E-25	4.00E-26±9.10E-26	1.04E-54±5.64E-54	1.43E-35±4.98E-35	**0.00E+00±0.00E+00**
***f***_**11**_	-5.00E-01±5.04E-01	**0.00E+00±0.00E+00**	8.51E-17±4.78E-17	7.40E-18±2.82E-17	1.52E-16±6.17E-17	**0.00E+00±0.00E+00**	**0.00E+00±0.00E+00**	**0.00E+00±0.00E+00**	**0.00E+00±0.00E+00**
***f***_**12**_	6.02E-27±3.16E-26	2.76E-48±7.81E-48	7.27E-48±1.23E-47	2.62E-35±7.06E-35	8.22E-38±2.21E-37	1.17E-67±2.19E-67	7.93E-99±4.01E-98	4.36E-55±1.59E-54	**0.00E+00±0.00E+00**
***f***_**13**_	5.69E-03±2.45E-03	**0.00E+00±0.00E+00**	**0.00E+00±0.00E+00**	1.75E-32±3.27E-33	**0.00E+00±0.00E+00**	**0.00E+00±0.00E+00**	2.21E+00±3.15E-01	4.62E+00±1.00E+00	6.17E-20±7.02E-20
***f***_**14**_	3.19E-01±2.34E-01	1.60E-03±1.20E-03	3.09E-03±1.45E-03	4.49E-03±2.21E-03	2.19E-03±1.09E-03	2.56E-03±1.53E-03	4.14E-01±2.81E-01	**1.48E-03±6.33E-04**	3.90E-03±2.04E-03
***f***_**15**_	2.58E+01±2.12E-01	7.46E-01±7.49E-01	7.41E-01±1.43E+00	1.29E+01±1.08E+00	**6.67E-01±1.51E+00**	9.52E+00±1.68E+00	2.80E+01±3.35E-01	2.89E+01±3.24E-02	2.56E+01±3.93E-01
***f***_**16**_	**0.00E+00±0.00E+00**	**0.00E+00±0.00E+00**	6.15E-03±1.06E-02	**0.00E+00±0.00E+00**	2.47E-04±1.35E-03	9.04E-04±2.86E-03	1.28E-03±4.96E-03	**0.00E+00±0.00E+00**	**0.00E+00±0.00E+00**
***f***_**17**_	4.38E+01±2.96E+01	1.10E+00±1.07E+00	5.85E-01±1.06E+00	9.09E+00±1.80E+00	2.43E+01±1.29E+01	9.05E+00±2.71E+00	3.88E-09±2.13E-08	6.09E+00±3.34E+01	**0.00E+00±0.00E+00**
***f***_**18**_	4.06E-14±1.55E-13	6.10E-03±1.29E-03	4.85E-16±5.14E-16	1.36E-02±6.64E-03	1.09E-12±5.90E-12	3.18E-15±1.57E-14	5.28E-51±1.36E-50	1.56E-25±4.48E-25	**0.00E+00±0.00E+00**
***f***_**19**_	**0.00E+00±0.00E+00**	**0.00E+00±0.00E+00**	3.99E-01±6.27E-01	7.40E-18±2.82E-17	2.04E-16±1.64E-16	2.75E-02±1.05E-01	**0.00E+00±0.00E+00**	**0.00E+00±0.00E+00**	**0.00E+00±0.00E+00**
***f***_**20**_	1.42E-01±4.78E-02	1.07E-01±2.54E-02	2.43E-01±5.04E-02	1.83E-01±3.79E-02	2.93E-01±6.91E-02	3.63E-01±8.50E-02	9.99E-02±1.21E-07	5.00E-02±5.08E-02	**0.00E+00±0.00E+00**
***f***_**21**_	3.09E+01±7.16E+00	2.53E+00±7.45E-01	2.69E+00±5.36E-01	3.70E+00±7.72E-01	1.29E+01±8.01E+00	1.24E+00±3.30E-01	6.12E-02±8.24E-02	1.69E+01±2.42E+01	**0.00E+00±0.00E+00**
***f***_**22**_	4.38E-15±3.89E-15	3.55E-15±0.00E+00	6.28E-15±1.53E-15	5.68E-15±1.77E-15	2.29E-14±6.31E-15	3.55E-15±0.00E+00	**0.00E+00±0.00E+00**	3.55E-15±0.00E+00	**0.00E+00±0.00E+00**
***f***_**23**_	**0.00E+00±0.00E+00**	1.10E-12±4.30E-12	1.09E-01±1.65E-01	**0.00E+00±0.00E+00**	8.88E-02±7.20E-02	**0.00E+00±0.00E+00**	**0.00E+00±0.00E+00**	**0.00E+00±0.00E+00**	**0.00E+00±0.00E+00**
***f***_**24**_	6.24E-01±1.04E-01	1.89E-02±3.73E-03	7.86E-03±2.32E-03	1.83E-02±1.73E-03	7.36E-02±4.85E-02	3.78E-02±4.31E-03	6.49E-01±1.05E-01	1.40E+00±1.16E+00	**0.00E+00±0.00E+00**
***f***_**25**_	4.55E-01±5.40E-02	**1.40E-01±3.66E-02**	2.30E-01±5.30E-02	2.19E-01±2.68E-02	2.38E-01±6.01E-02	2.53E-01±3.94E-02	8.32E-01±8.93E-02	5.80E-01±1.20E-01	3.59E-01±4.85E-02
***f***_**26**_	3.93E-01±3.92E-02	3.71E-01±8.88E-02	4.39E-01±1.94E-01	**3.48E-01±9.30E-02**	4.09E-01±1.33E-01	3.95E-01±1.51E-01	4.98E-01±5.87E-03	4.40E-01±3.62E-02	4.18E-01±2.93E-02
***f***_**27**_	**0.00E+00±0.00E+00**	**0.00E+00±0.00E+00**	**0.00E+00±0.00E+00**	**0.00E+00±0.00E+00**	1.48E-17±8.11E-17	**0.00E+00±0.00E+00**	**0.00E+00±0.00E+00**	**0.00E+00±0.00E+00**	**0.00E+00±0.00E+00**
***f***_**28**_	3.43E+00±4.78E-01	5.49E-01±9.74E-02	5.43E-01±7.57E-02	7.37E-01±8.86E-02	5.70E-01±6.53E-02	4.12E-01±9.71E-02	4.38E+00±1.12E+00	**0.00E+00±0.00E+00**	**0.00E+00±0.00E+00**
***f***_**29**_	1.21E+01±8.27E-01	**2.01E+00±2.70E-01**	2.23E+00±1.82E-01	2.80E+00±2.07E-01	2.95E+00±3.78E-01	3.18E+00±1.11E+00	1.15E+01±3.77E-01	1.32E+01±1.30E+00	7.67E+00±5.40E-01
***f***_**30**_	1.68E+01±8.02E+00	**0.00E+00±0.00E+00**	1.10E-07±6.02E-07	**0.00E+00±0.00E+00**	3.41E+01±1.34E+01	1.39E+01±4.21E+00	**0.00E+00±0.00E+00**	6.97E+00±2.69E+01	**0.00E+00±0.00E+00**
***f***_**31**_	2.61E-04±8.77E-05	**1.57E-32±5.57E-48**	**1.57E-32±5.57E-48**	**1.57E-32±5.57E-48**	2.78E-32±1.35E-32	**1.57E-32±5.57E-48**	6.99E-02±2.08E-02	2.52E-01±9.43E-02	1.04E-22±1.26E-22
***f***_**32**_	9.31E-03±3.99E-03	1.36E-31±2.77E-33	**1.35E-31±6.68E-47**	1.42E-31±1.24E-33	**1.35E-31±6.68E-47**	**1.35E-31±6.68E-47**	3.54E-01±8.20E-02	1.20E+00±3.83E-01	1.19E-21±2.02E-21

Best results are shown in bold

**Table 7:** Mean and STD obtained by eight enhanced DEs and DEGH on benchmark functions at 50D.

F	IMMSADE	CIPDE	EBDE	EDE	EJADE	LSHADE-SPACMA	DEPSO	ATLDE	DEGH
	Mean ± STD	Mean ± STD	Mean ± STD	Mean ± STD	Mean ± STD	Mean ± STD	Mean ± STD	Mean ± STD	Mean ± STD
***f***_**1**_	2.07E-20±1.03E-19	6.43E-34±1.54E-33	3.56E-25±6.06E-25	4.70E-20±1.41E-19	5.33E-21±9.49E-21	7.12E-36±1.63E-35	2.92E-95±1.57E-94	7.20E-54±1.18E-53	**0.00E+00±0.00E+00**
***f***_**2**_	1.19E-16±6.46E-16	7.56E-29±1.14E-28	1.26E-19±5.82E-19	1.68E-15±7.21E-15	4.17E-16±7.11E-16	3.11E-12±1.32E-11	2.25E-94±7.88E-94	2.30E-49±6.00E-49	**0.00E+00±0.00E+00**
***f***_**3**_	3.07E-15±1.63E-14	1.02E-27±2.17E-27	3.18E-17±9.51E-17	4.05E-12±1.48E-11	6.74E-14±2.25E-13	2.87E-24±9.59E-24	7.03E-87±3.85E-86	1.38E-47±3.17E-47	**0.00E+00±0.00E+00**
***f***_**4**_	5.77E+02±1.03E+03	3.32E-02±3.51E-02	8.06E-03±7.01E-03	3.73E+00±2.75E+00	4.96E-02±3.69E-02	2.48E-03±3.24E-03	1.56E-90±8.42E-90	1.59E-52±3.48E-52	**0.00E+00±0.00E+00**
***f***_**5**_	1.97E-12±7.76E-12	1.12E-17±1.10E-17	3.86E-13±7.37E-13	1.25E-12±1.66E-12	3.02E-08±4.42E-08	1.18E-23±6.48E-24	1.37E-51±6.72E-51	2.84E-26±5.51E-26	**0.00E+00±0.00E+00**
***f***_**6**_	2.30E-05±4.93E-05	3.72E-01±2.37E-01	6.26E-01±3.56E-01	1.65E+00±7.73E-01	1.70E+00±7.41E-01	1.07E-01±6.23E-02	5.06E-47±2.75E-46	8.70E-23±1.73E-22	**0.00E+00±0.00E+00**
***f***_**7**_	1.01E-65±5.47E-65	2.90E-27±1.58E-26	1.91E-21±9.86E-21	1.46E-06±6.55E-06	8.34E-33±2.25E-32	4.44E-25±1.80E-24	4.10E-115±2.25E-114	1.67E-113±6.21E-113	**0.00E+00±0.00E+00**
***f***_**8**_	2.68E-24±1.32E-23	9.36E-35±8.78E-35	1.44E-25±2.50E-25	4.52E-21±1.03E-20	2.19E-21±4.08E-21	2.42E-41±1.11E-40	2.26E-97±9.99E-97	5.40E-54±2.48E-53	**0.00E+00±0.00E+00**
***f***_**9**_	2.09E-23±1.12E-22	4.82E-33±7.82E-33	4.23E-24±1.19E-23	1.96E-19±6.00E-19	7.29E-20±1.52E-19	2.58E-26±6.25E-26	4.77E-95±2.61E-94	2.89E-53±1.03E-52	**0.00E+00±0.00E+00**
***f***_**10**_	2.14E-15±1.16E-14	3.12E-16±3.43E-16	4.37E-12±5.19E-12	3.72E-08±7.10E-08	5.97E-13±6.78E-13	8.96E-11±2.42E-10	4.43E-53±2.42E-52	1.80E-32±2.98E-32	**0.00E+00±0.00E+00**
***f***_**11**_	-5.00E-01±5.04E-01	9.25E-17±4.21E-17	1.44E-16±5.17E-17	1.22E-16±3.39E-17	6.22E-16±1.76E-16	**0.00E+00±0.00E+00**	**0.00E+00±0.00E+00**	**0.00E+00±0.00E+00**	**0.00E+00±0.00E+00**
***f***_**12**_	5.70E-17±3.08E-16	4.90E-32±1.11E-31	1.80E-24±2.24E-24	3.31E-19±4.44E-19	8.00E-21±2.01E-20	6.06E-47±1.83E-46	1.70E-98±9.11E-98	1.99E-54±4.98E-54	**0.00E+00±0.00E+00**
***f***_**13**_	3.62E-02±1.15E-02	**2.95E-32±1.23E-32**	3.48E-25±5.47E-25	2.06E-20±5.50E-20	2.27E-21±2.64E-21	5.75E-32±1.69E-32	6.31E+00±4.62E-01	9.53E+00±8.98E-01	2.55E-10±2.36E-10
***f***_**14**_	4.04E-01±2.45E-01	2.77E-03±1.52E-03	1.20E-02±5.62E-03	7.61E-03±3.30E-03	9.90E-03±3.75E-03	6.24E-03±2.86E-03	3.57E-01±2.41E-01	**1.42E-03±6.74E-04**	5.07E-03±2.53E-03
***f***_**15**_	4.62E+01±3.55E-01	3.32E+01±1.46E+01	3.98E+01±1.89E+01	4.52E+01±1.29E+01	**2.98E+01±1.13E+01**	3.71E+01±1.31E+01	4.81E+01±3.48E-01	4.89E+01±3.81E-02	4.62E+01±2.84E-01
***f***_**16**_	**0.00E+00±0.00E+00**	1.23E-03±3.22E-03	4.51E-03±6.47E-03	4.93E-04±1.88E-03	1.48E-03±3.40E-03	4.76E-03±6.72E-03	**0.00E+00±0.00E+00**	**0.00E+00±0.00E+00**	**0.00E+00±0.00E+00**
***f***_**17**_	1.01E+02±8.39E+01	3.04E+01±3.96E+00	4.85E+00±2.75E+00	5.06E+01±4.17E+00	7.13E+01±5.00E+01	1.79E+01±3.67E+00	3.32E-02±1.82E-01	**0.00E+00±0.00E+00**	**0.00E+00±0.00E+00**
***f***_**18**_	1.83E-10±5.52E-10	1.61E-02±7.54E-03	7.29E-12±1.83E-11	1.65E-09±4.57E-09	1.48E-04±7.40E-04	3.47E-08±7.60E-08	5.02E-47±2.74E-46	3.23E-26±5.24E-26	**0.00E+00±0.00E+00**
***f***_**19**_	**0.00E+00±0.00E+00**	5.31E-02±1.71E-01	2.40E+00±1.44E+00	1.59E-01±4.13E-01	6.38E-01±6.82E-01	1.10E+00±1.03E+00	**0.00E+00±0.00E+00**	**0.00E+00±0.00E+00**	**0.00E+00±0.00E+00**
***f***_**20**_	1.87E-01±3.36E-02	2.33E-01±4.79E-02	5.40E-01±1.10E-01	2.73E-01±4.50E-02	7.87E-01±1.20E-01	6.53E-01±1.01E-01	9.99E-02±1.61E-07	5.03E-02±5.04E-02	**0.00E+00±0.00E+00**
***f***_**21**_	4.29E+01±1.81E+01	1.33E+01±2.55E+00	1.18E+01±1.31E+00	1.51E+01±1.98E+00	3.15E+01±1.97E+01	5.76E+00±9.21E-01	9.00E-02±1.44E-01	7.70E-01±2.11E+00	**0.00E+00±0.00E+00**
***f***_**22**_	1.26E-12±5.58E-12	7.11E-15±0.00E+00	1.64E+00±3.02E-01	3.36E-11±8.02E-11	2.93E-02±1.60E-01	3.67E-15±6.49E-16	**0.00E+00±0.00E+00**	3.55E-15±0.00E+00	**0.00E+00±0.00E+00**
***f***_**23**_	3.11E-10±1.65E-09	3.16E-04±1.33E-03	2.69E+00±1.06E+00	4.70E-03±1.25E-02	1.08E+00±3.82E-01	6.68E-02±2.26E-01	**0.00E+00±0.00E+00**	**0.00E+00±0.00E+00**	**0.00E+00±0.00E+00**
***f***_**24**_	1.18E+00±1.33E-01	4.31E-02±6.88E-03	1.27E-02±6.58E-03	6.26E-02±6.31E-03	1.53E-01±6.56E-02	6.26E-02±7.72E-03	1.22E+00±1.52E-01	1.86E+00±1.81E+00	**0.00E+00±0.00E+00**
***f***_**25**_	6.34E-01±5.71E-02	**2.74E-01±5.82E-02**	4.64E-01±8.71E-02	3.00E-01±4.38E-02	4.15E-01±9.54E-02	4.29E-01±7.21E-02	9.98E-01±9.79E-02	7.17E-01±1.20E-01	4.97E-01±6.37E-02
***f***_**26**_	**4.37E-01±6.17E-02**	4.74E-01±1.15E-01	5.86E-01±2.60E-01	4.84E-01±1.34E-01	5.49E-01±1.92E-01	5.25E-01±2.45E-01	5.00E-01±3.82E-05	4.65E-01±3.60E-02	4.40E-01±2.81E-02
***f***_**27**_	**0.00E+00±0.00E+00**	**0.00E+00±0.00E+00**	7.40E-17±1.68E-16	1.48E-17±8.11E-17	1.11E-15±9.04E-16	**0.00E+00±0.00E+00**	**0.00E+00±0.00E+00**	**0.00E+00±0.00E+00**	**0.00E+00±0.00E+00**
***f***_**28**_	9.01E+00±7.50E-01	1.83E+00±2.76E-01	1.66E+00±1.94E-01	2.21E+00±2.53E-01	1.62E+00±2.41E-01	1.40E+00±1.57E-01	1.15E+01±2.53E+00	1.68E-16±9.22E-16	**0.00E+00±0.00E+00**
***f***_**29**_	2.65E+01±1.06E+00	6.35E+00±5.41E-01	**5.90E+00±5.59E-01**	7.60E+00±5.99E-01	7.67E+00±1.13E+00	6.63E+00±1.39E+00	2.09E+01±3.02E-01	2.29E+01±3.36E-02	1.90E+01±1.11E+00
***f***_**30**_	9.43E+01±3.36E+01	3.26E-05±1.58E-04	1.07E-03±4.15E-03	3.53E+00±1.14E+00	9.12E+01±4.08E+01	3.28E+01±7.36E+00	**0.00E+00±0.00E+00**	1.05E-07±5.72E-07	**0.00E+00±0.00E+00**
***f***_**31**_	9.47E-04±3.81E-04	**9.42E-33±1.39E-48**	8.27E-29±1.65E-28	5.24E-24±1.15E-23	2.08E-24±5.75E-24	**9.42E-33±1.39E-48**	1.68E-01±3.42E-02	5.00E-01±1.11E-01	1.41E-13±1.08E-13
***f***_**32**_	6.04E-02±1.82E-02	2.05E-31±7.30E-32	1.29E-27±3.02E-27	4.29E-23±5.07E-23	7.47E-22±3.19E-21	**1.45E-31±1.92E-33**	9.58E-01±1.61E-01	3.66E+00±6.65E-01	3.57E-12±3.28E-12

Best results are shown in bold

**Table 8:** Mean and STD obtained by eight enhanced DEs and DEGH on benchmark functions at 100D.

F	IMMSADE	CIPDE	EBDE	EDE	EJADE	LSHADE-SPACMA	DEPSO	ATLDE	DEGH
	Mean ± STD	Mean ± STD	Mean ± STD	Mean ± STD	Mean ± STD	Mean ± STD	Mean ± STD	Mean ± STD	Mean ± STD
***f***_**1**_	3.03E-18±8.83E-18	3.36E-15±2.32E-15	6.91E-08±7.44E-08	2.22E-06±3.59E-06	4.58E-07±8.46E-07	1.85E-04±2.37E-04	1.95E-95±8.97E-95	2.54E-53±5.85E-53	**0.00E+00±0.00E+00**
***f***_**2**_	1.67E-14±8.56E-14	5.43E-10±7.83E-10	3.98E-03±6.90E-03	2.31E-02±3.61E-02	2.62E-01±6.27E-01	2.01E+03±3.34E+03	1.43E-91±6.88E-91	4.00E-48±1.22E-47	**0.00E+00±0.00E+00**
***f***_**3**_	5.02E-11±1.93E-10	1.28E-08±1.23E-08	3.18E-01±6.01E-01	3.66E+01±1.64E+02	2.60E+00±5.91E+00	1.04E+03±1.40E+03	3.78E-92±1.88E-91	1.44E-47±3.00E-47	**0.00E+00±0.00E+00**
***f***_**4**_	1.04E+04±9.86E+03	2.36E+02±7.04E+01	2.37E+02±9.21E+01	8.66E+02±1.95E+02	3.06E+02±1.33E+02	5.77E+02±2.38E+02	1.98E-89±1.07E-88	5.20E-50±2.50E-49	**0.00E+00±0.00E+00**
***f***_**5**_	4.44E-09±2.17E-08	1.48E-07±2.39E-07	1.11E-03±1.72E-03	1.06E-03±2.16E-03	1.43E-02±2.40E-02	2.02E-06±1.07E-05	1.41E-46±7.70E-46	5.56E-27±4.77E-27	**0.00E+00±0.00E+00**
***f***_**6**_	1.01E-03±3.16E-03	8.53E+00±1.13E+00	1.53E+01±1.73E+00	9.68E+00±1.31E+00	1.26E+01±1.90E+00	9.38E+00±1.62E+00	1.40E-49±3.71E-49	1.13E-22±1.51E-22	**0.00E+00±0.00E+00**
***f***_**7**_	9.83E-51±5.34E-50	1.92E+33±1.03E+34	1.38E+42±6.75E+42	1.66E+52±9.09E+52	6.20E+24±2.11E+25	3.33E+28±1.83E+29	4.01E-110±1.49E-109	4.82E-115±1.45E-114	**0.00E+00±0.00E+00**
***f***_**8**_	5.21E-16±2.45E-15	2.03E-15±1.44E-15	2.73E-08±2.79E-08	4.37E-07±5.91E-07	2.49E-07±4.55E-07	4.14E-09±4.98E-09	5.34E-97±2.07E-96	7.95E-54±2.06E-53	**0.00E+00±0.00E+00**
***f***_**9**_	3.76E-18±1.55E-17	1.39E-14±1.04E-14	3.00E-07±4.65E-07	1.79E-06±2.28E-06	3.10E-06±5.36E-06	2.22E-03±4.04E-03	3.86E-95±1.47E-94	2.24E-52±5.15E-52	**0.00E+00±0.00E+00**
***f***_**10**_	2.52E-11±1.16E-10	1.27E-06±6.16E-07	2.37E-03±1.88E-03	1.99E-02±1.03E-02	5.39E-04±4.33E-04	5.09E-02±4.31E-02	6.72E-54±1.58E-53	3.58E-30±7.75E-30	**0.00E+00±0.00E+00**
***f***_**11**_	-5.00E-01±5.04E-01	2.66E-16±6.90E-17	3.19E-12±4.24E-12	7.66E-11±1.52E-10	1.50E-11±1.29E-11	3.59E-16±8.59E-17	**0.00E+00±0.00E+00**	**0.00E+00±0.00E+00**	**0.00E+00±0.00E+00**
***f***_**12**_	1.55E-13±6.31E-13	1.86E-13±1.92E-13	1.25E-08±1.12E-08	8.12E-07±1.18E-06	1.79E-07±1.81E-07	2.55E-12±4.47E-12	7.42E-99±2.33E-98	1.87E-51±8.03E-51	**0.00E+00±0.00E+00**
***f***_**13**_	5.01E-01±1.70E-01	**5.54E-15±7.33E-15**	6.33E-08±8.57E-08	8.20E-07±7.28E-07	4.89E-07±6.08E-07	2.20E-04±2.09E-04	1.77E+01±5.63E-01	2.24E+01±8.51E-01	9.23E-04±6.82E-04
***f***_**14**_	3.99E-01±2.34E-01	3.89E-02±1.17E-02	1.30E-01±3.10E-02	3.76E-02±1.00E-02	1.34E-01±3.56E-02	3.15E-02±1.44E-02	3.84E-01±2.38E-01	**1.47E-03±6.19E-04**	5.67E-03±4.66E-03
***f***_**15**_	9.67E+01±3.47E-01	1.57E+02±5.33E+01	2.45E+02±6.61E+01	2.60E+02±5.90E+01	1.48E+02±5.25E+01	1.71E+02±5.07E+01	9.84E+01±2.42E-01	9.89E+01±4.28E-02	**9.63E+01±2.34E-01**
***f***_**16**_	5.88E-16±3.07E-15	5.40E-03±1.26E-02	1.54E-02±2.23E-02	6.77E-03±1.78E-02	5.06E-03±1.38E-02	1.76E-02±2.08E-02	1.35E-03±7.42E-03	**0.00E+00±0.00E+00**	**0.00E+00±0.00E+00**
***f***_**17**_	1.12E+02±2.09E+02	1.81E+02±8.87E+00	9.62E+01±1.06E+01	2.54E+02±1.40E+01	1.28E+02±5.62E+01	4.22E+01±5.93E+00	**0.00E+00±0.00E+00**	**0.00E+00±0.00E+00**	**0.00E+00±0.00E+00**
***f***_**18**_	1.26E-08±2.97E-08	1.93E+00±3.09E+00	1.31E+01±1.16E+01	8.52E-04±1.15E-03	1.76E+00±2.59E+00	4.11E-02±1.98E-02	2.63E-48±1.43E-47	2.04E-26±3.54E-26	**0.00E+00±0.00E+00**
***f***_**19**_	**0.00E+00±0.00E+00**	3.94E+00±2.57E+00	7.30E+00±2.94E+00	7.22E+00±2.74E+00	5.65E+00±3.12E+00	1.61E+01±3.92E+00	**0.00E+00±0.00E+00**	**0.00E+00±0.00E+00**	**0.00E+00±0.00E+00**
***f***_**20**_	2.10E-01±3.02E-02	6.63E-01±9.64E-02	1.44E+00±2.64E-01	8.93E-01±1.11E-01	2.57E+00±3.13E-01	1.28E+00±2.36E-01	9.99E-02±9.01E-08	6.14E-02±4.86E-02	**0.00E+00±0.00E+00**
***f***_**21**_	3.74E+01±4.06E+01	8.12E+01±6.33E+00	5.29E+01±5.76E+00	7.97E+01±8.23E+00	6.39E+01±1.38E+01	2.98E+01±9.94E+00	1.85E-02±2.35E-02	6.05E-03±6.47E-03	**0.00E+00±0.00E+00**
***f***_**22**_	2.69E-09±1.42E-08	1.66E+00±3.23E-01	3.46E+00±6.09E-01	1.61E+00±2.88E-01	1.90E+00±2.27E-01	2.32E+00±3.51E-01	**0.00E+00±0.00E+00**	3.55E-15±0.00E+00	**0.00E+00±0.00E+00**
***f***_**23**_	5.09E-06±2.62E-05	3.04E+00±1.13E+00	2.28E+01±3.69E+00	2.94E+00±9.32E-01	1.37E+01±2.09E+00	2.51E+00±1.41E+00	**0.00E+00±0.00E+00**	**0.00E+00±0.00E+00**	**0.00E+00±0.00E+00**
***f***_**24**_	2.08E+00±1.53E-01	1.65E-01±1.86E-02	7.63E-02±1.35E-02	3.03E-01±3.02E-02	5.53E-01±1.77E-01	1.64E-01±1.90E-02	2.10E+00±1.76E-01	2.17E+00±2.11E+00	**0.00E+00±0.00E+00**
***f***_**25**_	8.62E-01±5.26E-02	4.97E-01±8.12E-02	5.94E-01±8.92E-02	**4.72E-01±7.18E-02**	6.18E-01±1.04E-01	6.70E-01±7.55E-02	1.14E+00±1.06E-01	8.72E-01±1.24E-01	7.32E-01±7.18E-02
***f***_**26**_	5.19E-01±7.04E-02	5.83E-01±1.76E-01	5.90E-01±2.75E-01	5.82E-01±2.00E-01	6.00E-01±2.33E-01	5.63E-01±2.46E-01	5.00E-01±6.76E-14	4.92E-01±7.42E-03	**4.80E-01±1.52E-02**
***f***_**27**_	**0.00E+00±0.00E+00**	1.70E-16±2.72E-16	1.98E-12±2.55E-12	6.72E-11±8.22E-11	2.77E-11±3.99E-11	2.89E-16±3.69E-16	**0.00E+00±0.00E+00**	**0.00E+00±0.00E+00**	**0.00E+00±0.00E+00**
***f***_**28**_	2.77E+01±1.70E+00	8.61E+00±5.41E-01	7.23E+00±5.74E-01	9.49E+00±8.67E-01	6.88E+00±6.23E-01	5.98E+00±5.64E-01	2.76E+01±1.06E+01	1.05E-10±5.46E-10	**0.00E+00±0.00E+00**
***f***_**29**_	6.54E+01±7.53E+00	2.49E+01±1.32E+00	2.68E+01±3.24E+00	2.85E+01±2.08E+00	2.83E+01±4.85E+00	**1.29E+01±2.23E+00**	4.39E+01±4.10E-01	4.59E+01±3.80E-02	4.56E+01±3.82E-01
***f***_**30**_	3.38E+02±1.26E+02	3.67E+01±4.74E+00	1.77E+01±3.83E+00	7.98E+01±1.14E+01	2.17E+02±8.27E+01	8.14E+01±1.40E+01	**0.00E+00±0.00E+00**	**0.00E+00±0.00E+00**	**0.00E+00±0.00E+00**
***f***_**31**_	4.51E-03±2.07E-03	**1.80E-19±1.33E-19**	1.04E-03±5.68E-03	2.68E-11±2.86E-11	1.04E-03±5.68E-03	1.58E-15±1.47E-15	3.64E-01±5.49E-02	8.34E-01±1.26E-01	6.01E-07±2.62E-07
***f***_**32**_	5.73E-01±1.30E-01	**4.51E-17±3.96E-17**	1.25E-02±1.87E-02	5.77E-10±6.91E-10	3.33E-03±5.17E-03	3.70E-04±2.03E-03	4.22E+00±8.46E-01	9.51E+00±5.48E-01	2.19E-03±5.88E-03

Best results are shown in bold

**Table 9:** The results of Wilcoxon’s signed-rank test at the 0.05significance level between DEGH and eight DE variants.

DEGH vs	*D* = 30	*D* = 50	*D* = 100
	+/−/≈	+/−/≈	+/−/≈
IMMSADE	26/2/4	27/2/3	29/1/2
CIPDE	19/8/5	24/7/1	27/5/0
EBDE	24/7/1	26/6/0	29/3/0
EDE	21/7/4	26/6/0	27/5/0
EJADE	24/8/0	26/6/0	29/3/0
LSHADE-SPACMA	21/8/3	24/6/2	27/5/0
DEPSO	26/0/6	25/0/7	24/1/7
ATLDE	25/1/6	25/1/6	24/1/7

At D = 30, from Table 6, the proposed DEGH gets the global optimal solution on functions *f*_1_~*f*_12_, *f*_16_~*f*_24_, *f*_27_, *f*_28_ and *f*_30_. For step function *f*_13_, CIPDE, EBDE, EJADE and LSHADE-SPACMA obtain global optimum. For noise function *f*_14_, ATLDE is the best. EJADE, CIPDE, EDE and CIPDE give optimal solutions for multimodal functions *f*_15_, *f*_25_, *f*_26_ and *f*_29_, respectively. For *f*_31_, CIPDE, EBDE, EDE and LSHADE-SPACMA are best. For *f*_32_, LSHADE-SPACMA finds the best solution. As can be seen from the Wilcoxon signed-rank test results of D = 30 in Table 9, DEGH is superior to IMMSADE, CIPDE, EBDE, ED, EJADE, LSHADE- SPACMA, DEPSO and ATLDE on 26,19,24,21,24,21,26,25 out of 32 functions, respectively.

At *D* = 50, it can also be seen from Table 7 that DEGH gives the global minimum on *f*_1_~*f*_12_, *f*_16_~*f*_24_, *f*_27_, *f*_28_ and *f*_30_. For *f*_13_ and *f*_25_, CIPDE is the best. ATLDE, EJADE, IMMSADE, EBDE and LSHADE-SPACMA obtain the optimal solutions of *f*_14_, *f*_15_, *f*_26_, *f*_29_ and *f*_32_, respectively. CIPDE and LSHADE-SPACMA perform better than other algorithms on *f*_31_. According to the test results of the 50-dimensional problems in Table 9, among the 32 functions, DEGH has 27,24,26,26, 24,25 and 25 items that are better than IMMSADE, CIPDE, EBDE, ED, EJADE, LSHADE- SPACMA, DEPSO and ATLDE, respectively.

At *D* = 100, from Table 8, DEGH is best except two multimodal functions *f*_13_, *f*_14_ and four multimodal functions *f*_25_, *f*_29_, *f*_31_, *f*_32_. For *f*_13_, *f*_31_ and *f*_32_, CIPDE gets optimal solutions. ATLDE, EDE, and LSHADE-SPACMA find the best solutions on *f*_14_, *f*_25_ and *f*_29_, respectively. From Table 9, DEGH outperforms IMMSADE, CIPDE, EBDE, ED, EJADE, LSHADE- SPACMA, DEPSO and ATLDE on 29,27,29,27,29,27,24,24 functions, respectively.

**Fig 6:**
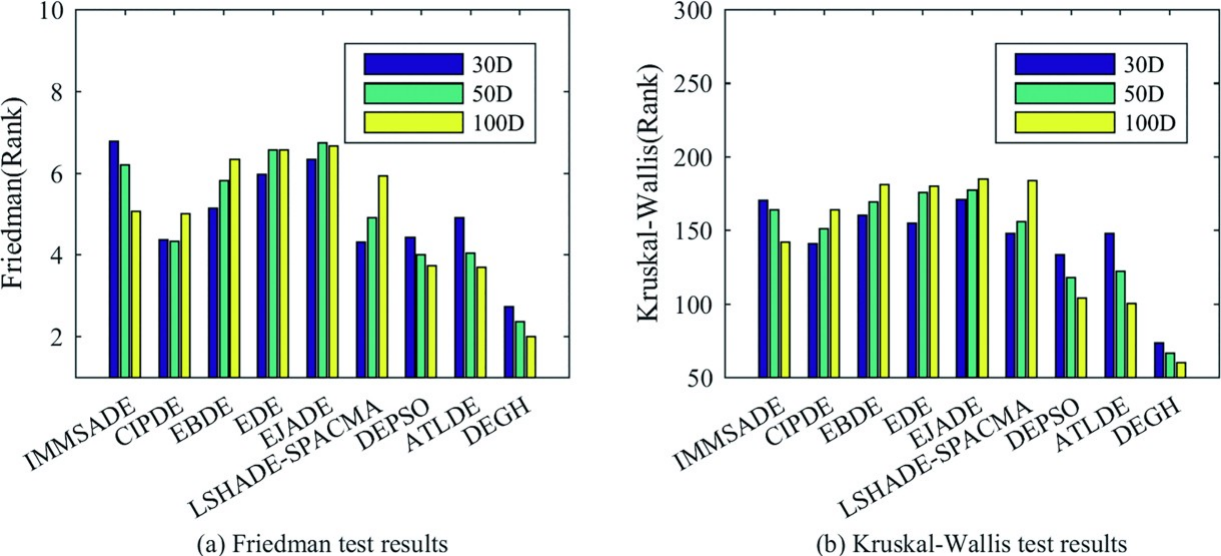
The results of the Friedman and Kruskal-Wallis tests for all algorithms at *D* = 30, 50, 100.

Furthermore, three non-parametric statistical tests are used to analyze these optimization results. The Friedman and Kruskal-Wallis tests results drawn in Fig 6 show that DEGH is the best in all dimensions. The Wilcoxon’s rank-sum test results in Table 10 show that all positive rank sums *R*^+^ obtained are far larger than negative rank sums *R*^−^, no matter in which dimension and compared with which algorithm. Moreover, whether the significance test level is 0.5 or 0.1, all p-values obtained are far less than them. In other words, Wilcoxon’s rank-sum test also confirms that DEGH is significantly superior to other compared algorithms.

**Table 10:** The Wilcoxon’s rank-sum test results for all algorithms at *D* = 30, 50, 100.

DEGH vs.	*D* = 30			*D* = 50			*D* = 100		
	*R*^+^	*R*^−^	*p-value*	*R*^+^	*R*^−^	*p-value*	*R*^+^	*R*^−^	*p-value*
IMMSADE	369	37	6.59e-05	398	37	4.44e-05	444	21	3.14e-05
CIPDE	235	143	3.63e-04	358	138	9.10e-06	449	79	3.44e-07
EBDE	359	137	1.31e-05	408	120	1.33e-06	478	50	9.01e-08
EDE	281	125	6.59e-05	419	109	1.24e-06	462	66	1.05e-07
EJADE	371	157	3.63e-06	416	112	8.15e-07	481	47	5.68e-08
LSHADE-SPACMA	282	153	8.84e-05	357	108	8.94e-06	466	62	5.68e-08
DEPSO	351	0	2.92e-04	325	0	5.43e-04	305	20	6.71e-04
ATLDE	337	14	2.10e-04	335	16	4.05e-04	310	15	9.62e-04

#### 4.3.2 Convergence properties

**Fig 7:**
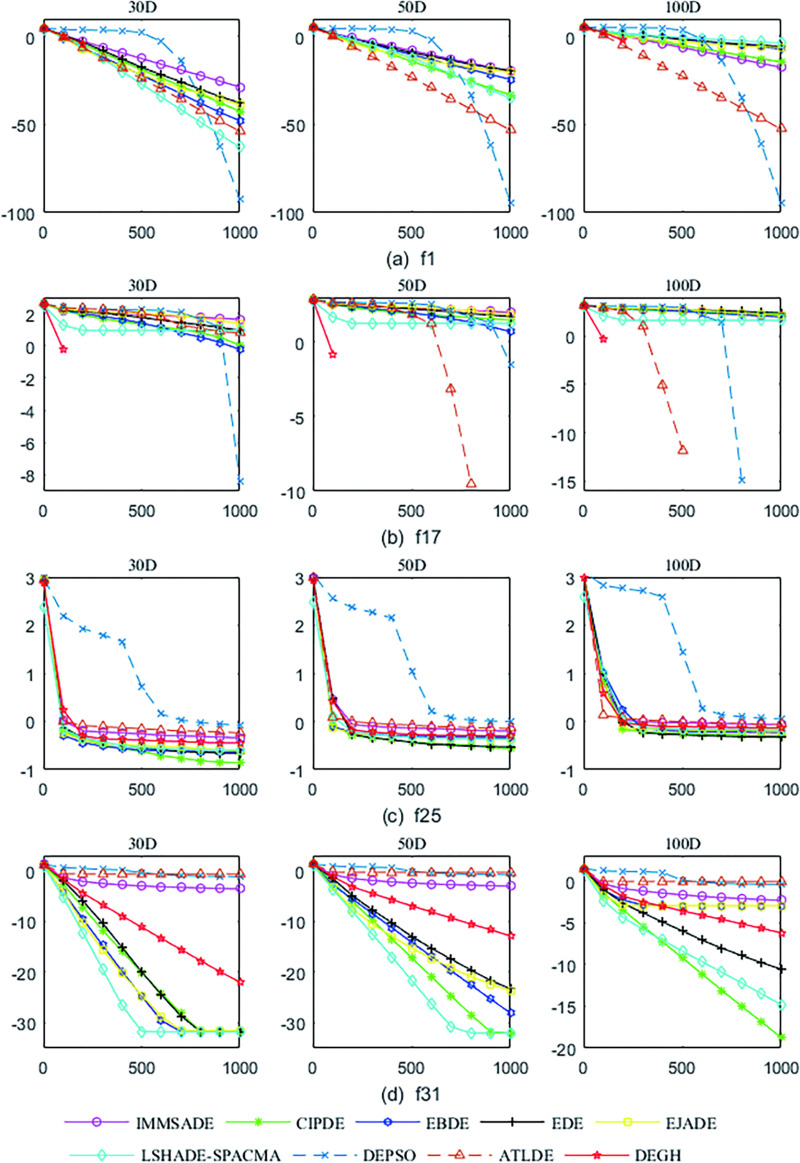
Convergence curves of the mean function error values for *f*_1_, *f*_17_, *f*_25_ and *f*_31_ at *D* = 30, 50, 100. The horizontal axis and the vertical axis are generations and the mean function error values over 30 independent runs.

The convergence properties can be summarized into the following four types, which are depicted in Fig 7.

- The convergence curves of *f*_1_~*f*_12_, *f*_16_, *f*_22_ and *f*_27_ are similar, as shown in Fig 7(A). In this type, DEGH does not show apparent advantages at the beginning of evolution, but it can quickly converge to the global minimum first.
- The convergence attributes of *f*_17_~*f*_21_, *f*_23_, *f*_24_, *f*_28_ and *f*_30_ are divided into a class, as shown in Fig 7(B). In this type, DEGH shows absolute advantages at the beginning, with the steepest slope and can quickly converge to the global minimum, while other algorithms evolve slowly or stall.
- In Fig 7(C), the convergence curve of *f*_25_ is plotted, which is similar to that of *f*_14_, *f*_15_, *f*_26_ and *f*_29_. On these functions, all algorithms are at varying degrees of evolutionary stagnation or slow evolution.
- The evolutionary trend of *f*_13_, *f*_31_ and *f*_32_ is similar, as shown in Fig 7(D). Here, some algorithms fall into evolutionary stagnation, but DEGH continues to evolve downward.

### 4.4 Discussion on results

The above experiments prove the remarkable superiority of the proposed DEGH. The reasons for DEGH’s outstanding performance are summarized as follows. (1) DEGH, based on GSK and HHO algorithms, is an improvement and hybrid on the DE framework. On the one hand, GSK/J-mutation and GSK/S-mutation operators have a good balance between global exploration and local exploitation. On the other hand, the DE/rand/1-mutation and HHO/SB-mutation are another powerful guarantee for the balance between exploration and exploitation. These two respects cooperate each other, formed the dual-safeguard mechanism for the balance between exploration and exploitation. (2) The crossover probability self-adaption strategy of DEGH strengthens the internal connection between the mutation, crossover and selection stages, and makes the whole frame structure more harmonious. On this basis, the crossover probability and scaling factor dynamically adjust the evolution strategy of each individual to make the proposed algorithm more suitable for various problems.

## 5 Conclusions

This paper proposes a hybrid differential evolution algorithm based on gaining-sharing knowledge algorithm and harris hawks optimization (DEGH), which can achieve excellent performance even with a fixed scaling factor. Through a series of experiments, the effectiveness and sensitivity of DEGH parameters are investigated. The performance of DEGH is evaluated by comparing with eight state-of-the-art DE variants like IMMSADE [19], CIPDE [20], EBDE [21], EDE [21], EJADE [30], LSHADE-SPACMA [35], DEPSO [37] and ATLDE [39] on 32 benchmark functions at D = 30,100. Experiments results show that: 1) DEGH is not sensitive to population size NP; 2) DEGH is insensitive to *F* except *F* = 0.1; 3) For all compared DE variants, DEGH has the best overall performance.

As an extension of this research work, the following aspects are the future research directions. 1) Binary version of DEGH and its application in flight sequencing system; 2) Apply DEGH to the optimization of neural network parameters and further apply it to flight trajectory prediction. 3) Hybridize DE with other emerging meta-heuristic algorithms.
